# Nocturnal mosquito Cryptochrome 1 mediates greater electrophysiological and behavioral responses to blue light relative to diurnal mosquito Cryptochrome 1

**DOI:** 10.3389/fnins.2022.1042508

**Published:** 2022-11-30

**Authors:** David D. Au, Jenny C. Liu, Thanh H. Nguyen, Alexander J. Foden, Soo Jee Park, Mia Dimalanta, Zhaoxia Yu, Todd C. Holmes

**Affiliations:** ^1^Department of Physiology and Biophysics, School of Medicine, University of California, Irvine, Irvine, CA, United States; ^2^Department of Statistics, Donald Bren School of Information and Computer Sciences, University of California, Irvine, Irvine, CA, United States; ^3^Center for Neural Circuit Mapping, School of Medicine, University of California, Irvine, Irvine, CA, United States

**Keywords:** cryptochrome, non-image forming vision, electrophysiology, light-evoked behavior, mosquito sensory biology, *Drosophila melanogaster*, *Anopheles gambiae*, *Aedes aegypti*

## Abstract

Nocturnal Anopheles mosquitoes exhibit strong behavioral avoidance to blue-light while diurnal Aedes mosquitoes are behaviorally attracted to blue-light and a wide range of other wavelengths of light. To determine the molecular mechanism of these effects, we expressed light-sensing Anopheles gambiae (AgCRY1) and Aedes aegypti (AeCRY1) Cryptochrome 1 (CRY) genes under a crypGAL4-24 driver line in a mutant Drosophila genetic background lacking native functional CRY, then tested behavioral and electrophysiological effects of mosquito CRY expression relative to positive and negative CRY control conditions. Neither mosquito CRY stops the circadian clock as shown by robust circadian behavioral rhythmicity in constant darkness in flies expressing either AgCRY1 or AeCRY1. AgCRY1 and AeCRY1 both mediate acute increases in large ventral lateral neuronal firing rate evoked by 450 nm blue-light, corresponding to CRY’s peak absorbance in its base state, indicating that both mosquito CRYs are functional, however, AgCRY1 mediates significantly stronger sustained electrophysiological light-evoked depolarization in response to blue-light relative to AeCRY1. In contrast, neither AgCRY1 nor AeCRY1 expression mediates measurable increases in large ventral lateral neuronal firing rates in response to 405 nm violet-light, the peak of the Rhodopsin-7 photoreceptor that is co-expressed in the large lateral ventral neurons. These results are consistent with the known action spectra of type 1 CRYs and lack of response in cry-null controls. AgCRY1 and AeCRY1 expressing flies show behavioral attraction to low intensity blue-light, but AgCRY1 expressing flies show behavioral avoidance to higher intensity blue-light. These results show that nocturnal and diurnal mosquito Cryptochrome 1 proteins mediate differential physiological and behavioral responses to blue-light that are consistent with species-specific mosquito behavior.

## Introduction

Many insect behaviors are modulated by short wavelength light (Coombe, 1982; Green and Cosens, 1983; Sumba et al., 2004; Das and Dimopoulos, 2008; Yamaguchi et al., 2010; Rund et al., 2012; Sawadogo et al., 2013; Tokushima et al., 2016; Knop et al., 2017; Sheppard et al., 2017; Farnesi et al., 2018; Padilha et al., 2018; Alonso San Alberto et al., 2022). It has been long assumed that insect behavioral light responses rely on image forming vision through eye photoreceptors that express opsins. However, insects additionally have non-image forming vision mediated by photoreceptors that are expressed directly in brain neurons (Fogle et al., 2011; Ni et al., 2017).

Insect non-imaging forming visual photoreceptors include ultraviolet, blue, and red-light activated Cryptochrome (CRY) that was first characterized as the primary circadian photoreceptor in *Drosophila* (Emery et al., 1998; Stanewsky et al., 1998) and violet-light activated Rhodopsin 7 (Rh7, Ni et al., 2017). Rh7 is an opsin photoreceptor expressed in central brain neurons that couples to G protein signaling pathways and also regulates light-evoked circadian photo-attraction/avoidance behaviors (Ni et al., 2017; Kistenpfennig et al., 2018; Baik et al., 2019b; Lazopulo et al., 2019). CRY is a riboflavin-based photoreceptor protein that uses flavin adenine dinucleotide (FAD) as its light sensing chromophore. In *Drosophila*, CRY is expressed in roughly half of all circadian neurons (Emery et al., 2000; Klarsfeld et al., 2004; Benito et al., 2008; Sheeba et al., 2008c; Yoshii et al., 2008; Fogle et al., 2011), which include all of the Pigment Dispersing Factor (PDF) expressing ventral lateral neurons (LNvs) that also mediate light-evoked behavioral arousal (Parisky et al., 2008; Shang et al., 2008; Sheeba et al., 2008c,^2010^; Liu et al., 2014; Fogle et al., 2015; Muraro and Ceriani, 2015; Buhl et al., 2016; Potdar and Sheeba, 2018; Chaturvedi et al., 2022). While *Drosophila* only express light sensitive “type 1” CRYs, other insects also express light insensitive “type 2” CRYs similar to CRYs expressed in vertebrates that function as transcriptional repressors (Yuan et al., 2007; Gegear et al., 2010; Fogle et al., 2011; Damulewicz and Mazzotta, 2020). The best characterized function of CRYs in insects is the light activated initiation of the slow (∼1 h) and irreversible process of circadian clock resetting that has been well characterized by molecular genetic analysis in *Drosophila*. This mechanism occurs by CRY mediated light activated protein degradation of the heteromultimeric clock protein complex consisting of TIMELESS (TIM), PERIOD (PER), and CRY itself, thus relieving repression of the transcriptional activators CLOCK and CYCLE at E-box promoter sequences upstream from the *tim* and *per* genes (Emery et al., 1998; Stanewsky et al., 1998; Busza et al., 2004; Koh et al., 2006; Peschel et al., 2009; Damulewicz and Mazzotta, 2020).

CRY photoactivation also evokes rapid and very long-lasting (30–40 s) neuronal depolarization and increased spontaneous action potential firing in large ventral lateral neurons (l-LNvs) and other CRY expressing neurons (Sheeba et al., 2008b; Fogle et al., 2011, 2015; Giachello et al., 2016; Baik et al., 2017, 2019a; Hong et al., 2018; Au et al., 2022). While light-evoked CRY mediated electrophysiological effects are acute and reversible in contrast to CRY mediated clock resetting, CRY on/off electrophysiological kinetic light responses are not as rapid as those mediated by image-forming opsins. Light-activated CRY couples to electrophysiological depolarization and clock resetting through multiple mechanisms including photoreduction electron transfer events along a chain of CRY tryptophan residues in close proximity to the FAD chromophore and CRY protein conformational changes, including the C terminal tail (Berndt et al., 2007; Bouly et al., 2007; Hoang et al., 2008; Öztürk et al., 2008; Liu et al., 2010; Ozturk et al., 2011, 2014; Vaidya et al., 2013; Fogle et al., 2015; Lin et al., 2018, 2022; Baik et al., 2019a; Chandrasekaran et al., 2021). In addition to circadian clock resetting, CRY phototransduction evokes acute behaviors in insects, including arousal (Sheeba et al., 2008a; Fogle et al., 2015) and short wavelength light attraction/avoidance behavior (Baik et al., 2017, 2018, 2019b,^2020^; Au et al., 2022), which is under circadian modulation.

Light-activated CRY evoked behavioral changes are particularly interesting in mosquitoes as mosquito-spread diseases afflict hundreds of millions of people worldwide. Two medically important genera include nocturnal *Anopheline* and diurnal *Aedes* mosquitoes. *Anopheline* mosquitoes are responsible for over 200 million cases of malaria worldwide. *Aedes* mosquitoes are the principal vectors for Dengue virus (over 90 million cases worldwide) and yellow fever, West Nile fever, chikungunya fever, Zika fever, and Japanese encephalitis (WHO website fact sheet). Insect control methods based on the sensory physiology of mosquitoes is very appealing as chemical pesticides are non-specific and environmentally harmful. The behavior of nocturnal *An. gambiae* (Ag) and diurnal *Ae. aegypti* (Ae) mosquitoes is subject to circadian regulation, thus enforcing their ecologically distinct temporal activity patterns (Jones et al., 1967; Taylor and Jones, 1969). Recently, we found that nocturnal *An. coluzzii* and diurnal *Ae. aegypti* mosquitoes display distinct innate circadian temporal attraction/avoidance behavioral responses to light. Nocturnal *Anopheles* mosquitoes behaviorally avoid short wavelength light during the day, while diurnal *Aedes*, particularly females, are behaviorally attracted to a broad range of light spectra during the day (Baik et al., 2020). Attraction/avoidance behavioral responses to light for both species change with time-of-day and show distinct sex differences that are consistent with predation and mate swarming activities of females vs. males. These distinct *Anopheles* and *Aedes* mosquito behavioral light responses appear to be mediated by light activated type 1 Cryptochrome signaling shown by disruption of these behaviors by prior exposure to constant light (Baik et al., 2020). Further, attraction/avoidance behavioral responses to light are mediated by ventral lateral neurons that are characterized by PDF and PER proteins co-expressed in *Drosophila melanogaster* and other insect species. We recently showed that *Ae. aegypti* and *An. coluzzii* mosquito female adult brains also display characteristics of large- (l-LNvs) and small-ventral lateral neurons (s-LNvs) marked by PDF and PER co-expression with similar morphology and projection patterning (Baik et al., 2020). Putative circadian dorsal neurons (DNs) are seen in both *Ae. aegypti* and *An. coluzzii* mosquito female adult brains, again identified by similar morphological projections in common with *Drosophila* (Baik et al., 2020). Therefore, we employed an “empty-neuron” model approach using transgenic *Drosophila* on a *cry-null* background to express AgCRY1 and AeCRY1. In that paper we show mosquito CRY electrophysiological and behavioral responses to UV and red-light and find by multiple assays that nocturnal AgCRY1 is significantly more light sensitive as compared with diurnal AeCRY1. In Au et al. (2022) we focused on those two light wavelengths because UV light is the most commonly used part of the light spectrum for insect control devices using light (“bug lights”) to trap mosquitoes. We earlier characterized nocturnal and diurnal mosquito behavioral responses to UV light (Baik et al., 2020). Red light is of interest because we found distinctly different nocturnal and diurnal mosquito behavioral responses to red light (Baik et al., 2020). This followed our unexpected findings that insect CRYs functionally respond to red light (Baik et al., 2019a), in contrast to the lack of response of purified insect CRYs to red light for *in vitro* biophysical assays. In addition to CRYs which show spectral absorbance peaks in their base oxidized states to 365 nm UV light and 450 nm blue light, another photoreceptor, Rhodopsin 7 (Rh7) is expressed in the LNv and other brain neurons (Ni et al., 2017; Kistenpfennig et al., 2018; Baik et al., 2019b). Rh7 exhibits a comparatively broad spectral absorbance that peaks around 405 nm violet light. To compare the potential interactions between mosquito CRYs and Rh7, we tested AgCRY1 and AeCRY1 expressing transgenic flies for their responses to 450 nm blue light and 405 nm violet light.

## Materials and methods

### Experimental animals

*Drosophila melanogaster* flies were raised on standard media (yeast, cornmeal, agar) at 25 ± 1°C and 40–60% relative humidity in 12:12 h Light:Dark cycles. All flies used in experiments were first isogenized (backcrossed) to the w1118 genetic background for a minimum of six generations. All behavioral experiments used 3–4-day post-eclosion adult male flies. We generated pJFRC7 vectors containing cryptochrome 1 from *Drosophila melanogaster* (Dm), *An. gambiae* (Ag), and *Ae. aegypti* (Ae) in frame with eGFP. Use of the pJFRC7 vector allows for a controlled site-specific PhiC31 genomic insertion site. DNA constructs were then sent to the vendor Bestgene for fly embryonic injection and screening for successful transgenesis. Experimental transgenic flies backcrossed to the common wild-type w1118 background for a minimum of 6 generations. Genotyping primers were designed with the following sequences: AeCRY1 Forward: CGA GAA AGT GCA GGC CAA CAA TC, AeCRY1 Reverse: GT TCT TCA ACT CCG GCA GAT ATC, AgCRY1 Forward: CAG CCA GTT CAA GTA TCC GG, and AgCRY1 Reverse: CGG TTC GTG CAC AAA CTG TG. Experimental transgenic flies were crossed with a *cry-null* background (obtained from Jeff Hall, Brandeis University), then with a *crypGAL4-24* driver line for CRY-neuron specific expression of DmCRY or mosquito CRY1.

### Locomotor activity behavioral assay

Adaptations to the behavioral assays from Nitabach et al. (2002), Chiu et al. (2010), and Nave et al. (2021) were made for testing constant dark conditions for circadian behavior following 12 h:12 h light:dark entrainment (LD:DD) tested under two light intensities of l lux and 400 lux white light. Adult male flies (2–4 days post-eclosion) were anesthetized over CO_2_ and individually loaded into borosilicate activity tubes. The TriKinetics Locomotor Activity Monitoring System was used to track fly behavior over a protocol of: 12:12 h Light:Dark (LD) entrainment for 7 days, then 7 days of constant dark (DD) conditions. Actograms were generated using Clocklab software. Average activity education graphs and its statistics were measured using FaasX software, then graphed using Microsoft Excel. Within FaasX, the CycleP analysis toolkit was used to calculate % rhythmicity from periodogram analysis with the following scoring criteria for flies in DD: minimum power ≥ 20, minimum width (h) ≥ 2, Chi-square significance ≥ 0.05 and calculation of tau. Data are reported as averages ± standard error mean. Anticipation index measurements during LD were adapted for the entrainment duration from Harrisingh et al. (2007) and Sheeba et al. (2010) taking the average activity in the 3 h preceding lights on (morning anticipation) or lights off (afternoon/evening anticipation) as a ratio over the average activity in the 6 h preceding lights on or off for individual flies over 5 days of LD entrainment. The reported values for anticipation index are an average of all the flies over the 5 days of LD entrainment.

### Immunocytochemistry

**FIGURE 1:**
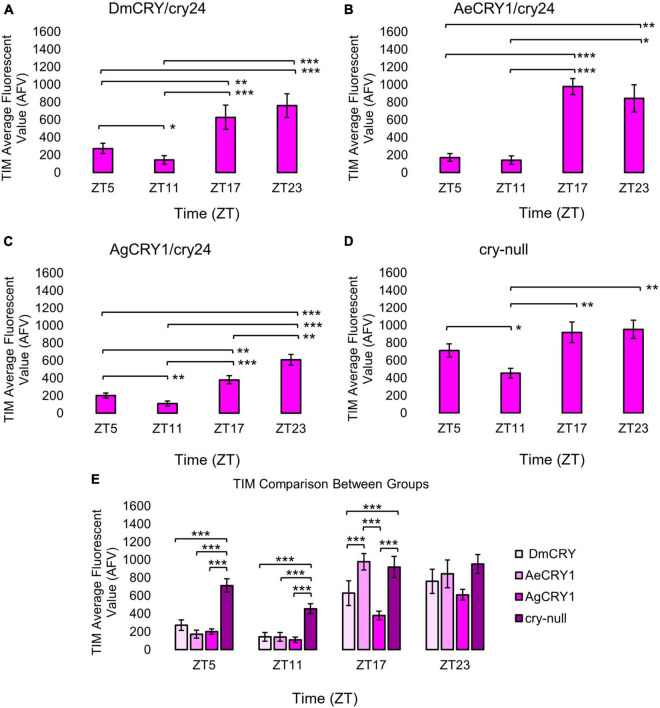
Transgenic mosquito CRY1 expression does not alter the overall pattern of cyclic TIM expression. Immunocytochemistry average fluorescent value of TIM expression over 12:12 h LD at ZT5, 11, 17, and 23 time points in LNvs (small + large) expressing **(A)** DmCRY (ZT5, *n* = 38; ZT11, *n* = 26; ZT17, *n* = 29; ZT23, *n* = 33), **(B)** AeCRY1 (ZT5, *n* = 15; ZT11, *n* = 10; ZT17, *n* = 26; ZT23, *n* = 26), **(C)** AgCRY1 (ZT5, *n* = 29; ZT11, *n* = 27; ZT17, *n* = 37; ZT23, *n* = 44), and **(D)** negative control *cry-null* (ZT5, *n* = 41; ZT11, *n* = 26; ZT17, *n* = 29; ZT23, *n* = 52). Fluorescent quantification of TIM signal was obtained by marking regions-of-interest on LNv soma identified by morphology and anatomical positioning within each brain sample. Fluorescent values for the total number of neurons in a brain are normalized to the background brain fluorescence, then measurements of all neurons from all brain samples are averaged together. **(E)** Comparison summary between genotypes for each time point measurement of average TIM fluorescence. Mann-Whitney *U*-tests with FDR adjustment were performed for statistical comparison. Data are represented as mean ± SEM for. *p ≤ 0.1, **p ≤ 0.05, ***p ≤ 0.01.

**FIGURE 2:**
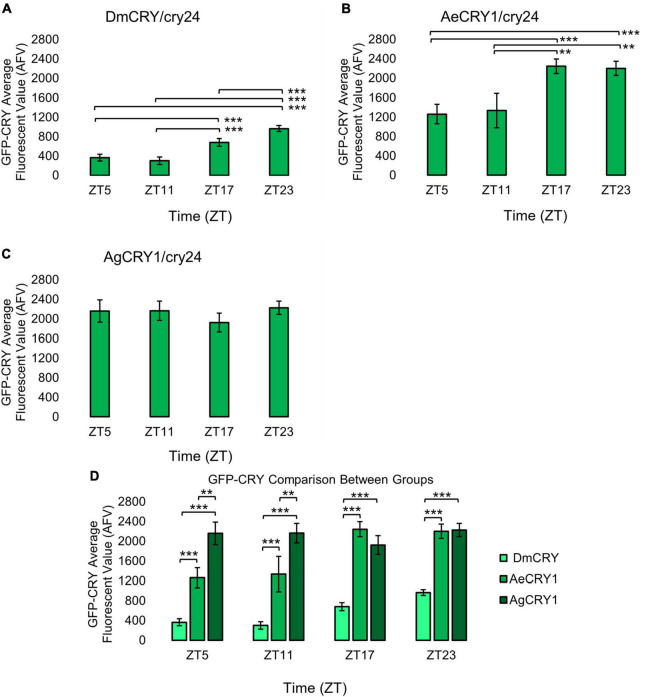
AeCRY1 and DmCRY shows lower protein levels during day and higher GFP-CRY during night, while AgCRY1 expression remains high throughout all time points. Immunocytochemistry average fluorescent value of GFP-CRY expression over 12:12 h LD at ZT5, 11, 17, and 23 time points in LNvs (small + large) expressing **(A)** DmCRY (ZT5, *n* = 38; ZT11, *n* = 26; ZT17, *n* = 29; ZT23, *n* = 33), **(B)** AeCRY1 (ZT5, *n* = 15; ZT11, *n* = 10; ZT17, *n* = 26; ZT23, *n* = 26), and **(C)** AgCRY1 (ZT5, *n* = 29; ZT11, *n* = 27; ZT17, *n* = 37; ZT23, *n* = 44). Fluorescent quantification of GFP-CRY signal was obtained by marking regions-of-interest on LNv soma identified by morphology and anatomical positioning within each brain sample. Fluorescent values for the total number of neurons in a brain are normalized to the background brain fluorescence, then measurements of all neurons from all brain samples are averaged together. **(D)** Comparison summary between genotypes for each time point measurement of average GFP-CRY fluorescence. Mann-Whitney *U*-tests with FDR adjustment were performed for statistical comparison. Data are represented as mean ± SEM. *p ≤ 0.1, **p ≤ 0.05, ***p ≤ 0.01.

Experimental transgenic flies were dissected for *ex vivo* brain preparations after 3–5 days of 12:12 h LD entrainment. Dissections began approximately 1 h before each ZT time point measured (ZT5, 11, 17, 23). Immunocytochemistry (ICC) experiments were performed for all genotypes in a given experiment, then repeated a minimum of 3 times to optimize statistical analysis and minimize experimental error. Dissected brains were placed in chilled 1X PBS, fixed in 4% paraformaldehyde (PFA) for 30 min, washed 3 × 10 min in PBS-Triton-X 1%, incubated in blocking buffer (10% Horse Serum-PBS-Triton-X 0.5%) at room temperature before incubation with rabbit α-TIM, polyclonal (1:1,000) antibodies overnight in 4°C. 3 rinse steps were performed at 10 min intervals with PBS-Triton-X 0.5% then incubated in goat α-rabbit-Alexa- 594 (1:1,000) secondary antibodies in blocking buffer overnight in 4°C. Brains were then rinsed 5 times at 15 min intervals in PBS-Triton-X 0.5% before mounting in Vectashield mounting media (Vector Laboratories). Sample slides were imaged using a Leica SP8 confocal microscope. We reproduced the TIM and CRY-GFP experiments published in Au et al. (2022), Current Biology and pooled the data with the earlier data for the results and updated total *n*’s reported in Figures 1, 2. The *n*’s for the new data added to the earlier data are: for ZT 5: DmCRY: 7, AeCRY1: 4, AgCRY1: 9, *cry-null*: 7; for ZT 11: DmCRY: 10, AeCRY1: 4, AgCRY1: 7, *cry-null*: 7; for ZT 17: DmCRY: 4, AeCRY1: 16, AgCRY1: 15, *cry-null*: 12; for ZT 23: DmCRY: 10, AeCRY1: 14, AgCRY1: 22, *cry-null*: 18.

### Confocal microscopy and image processing

For the data in Figures 1, 2, brain samples were imaged with a Leica SP8 confocal microscope with 594 nm antibody fluorescence for TIM signal and 488 nm CRY-GFP signal. FIJI/ImageJ analysis software was utilized for quantification of ventral lateral neuronal. Maximum intensity projections were generated using the Z stack tool. Fluorescent quantification of TIM and CRY-GFP signal were obtained by marking regions-of-interest on LNv (small and large LNvs) soma identified by morphology and anatomical positioning within each brain sample. Fluorescent values for the total number of neurons in a brain are normalized to the background brain fluorescence, then measurements of all neurons from all brain samples are averaged together.

### Light-evoked neuronal electrophysiology

Previously established whole-cell current-clamp protocols from Baik et al. (2019a) were modified to run our light-evoked potential electrophysiology experiments. Adult male fly brains were dissected in external recording solution consisting of: 122 mM NaCl, 3 mM KCl, 1.8 mM CaCl_2_, 0.8 mM MgCl_2_, 5 mM glucose, 10 mM HEPES, 7.2 pH, and calibrated to an osmolarity of 250–255 mOsm. The internal recording solution consists of: 102 mM Kgluconate, 17 mM NaCl, 0.085 mM CaCl_2_, 1.7 mM MgCl_2_ (hexahydrate), 8.5 mM HEPES, 0.94 mM EGTA, 7.2 pH, and is calibrated to an osmolarity of 232–235 mOsm. Custom multichannel LED source (Prizmatix/Stanford Photonics, Palo Alto, CA, United States) fitted to the Olympus BX51 WI microscope was used as the primary light source for our electrophysiology experiments. LED peak wavelengths are as follows: UV (365 nm), violet (405 nm), blue (450 nm), and red (635 nm), and all exposures were set to an intensity of 200 μW/cm^2^ by use of a Newport 842-PE Power/Energy meter. Each LED was triggered on and off for each sweep with TTL pulses programmed by pClamp (Molecular Dynamics) data acquisition software. The light-evoked potential protocol is as follows: 50 s of dark for baseline recording, 5 s of colored-light stimulation, then 95 s of inter-pulse darkness for recovery back to baseline. The protocol repeats five times per recording. For analysis, sweeps are averaged, and baseline adjusted to pre-pulse signal, then low-pass noise filtered using Gaussian and Butterworth filters in the ClampFit 10 software (Molecular Dynamics). Our light-evoked potential protocol captures averaged light-evoked changes in membrane potential (Fogle et al., 2011; Baik et al., 2019a; Au et al., 2022), thus providing a kinetically robust light-evoked potential.

### Light attraction/avoidance behavioral assay

Standard LD light choice assays were conducted using behavioral protocols developed in previous studies (Baik et al., 2017; Au et al., 2022). The locomotor activity of individual flies was measured using the TriKinetics Locomotor Activity Monitoring System via dual infrared beam-crossing, recording total crosses in 1-min bins. Individual flies housed on glass tubes have a choice of exposure to a lighted side or in a dark side blocked by aluminum foil of the two infrared sensor tube. Percentage activity and statistics were measured using Microsoft Excel. Custom LED fixtures were built using Waveform Lighting blue and red LEDs with a narrow peak wavelength of 450 and 405 nm, respectively, and intensity-tuned to 10 and 400 μW/cm^2^ for low and high intensity light exposures, respectively.

### Quantification and statistical analysis

All reported values are represented as mean ± SEM. Values of n refer to the total number of experimental flies tested over all replicates of an experiment (minimum of three replicates). Firing frequency values are calculated as a ratio of spikes during the 5 s of lights on/average baseline firing rate binned in 10 s increments. Statistical tests were performed using Minitab, Matlab, and Microsoft Excel software. Statistical analysis began with performing an Anderson-Darling normality tests to determine normality of data. Variance was determined using *F*-tests for normally distributed data, then significance was determined using two-sample, one-tailed *T*-tests with alpha values of 0.5 before pairwise correction. Significance for non-normal data was determined by Mann-Whitney *U*-tests. Spike firing and membrane potential quantifications were performed using custom Matlab scripts and Clampfit software. Multi-comparison tests leading to Type I error/false positives were mitigated by a more stringent test of *p*-value adjustment based on false discovery rate (FDR, Benjamini and Hochberg, 1995, see also Au et al., 2022). A standard FDR threshold of 0.1 was then implemented in order to indicate significance as an expected proportion of false positives that is no greater than 10%.

## Results

### *Anopheles gambiae* and *Aedes aegypti* expression is not sufficient to alter diurnal/nocturnal behavior or stop circadian rhythmicity

**FIGURE 3:**
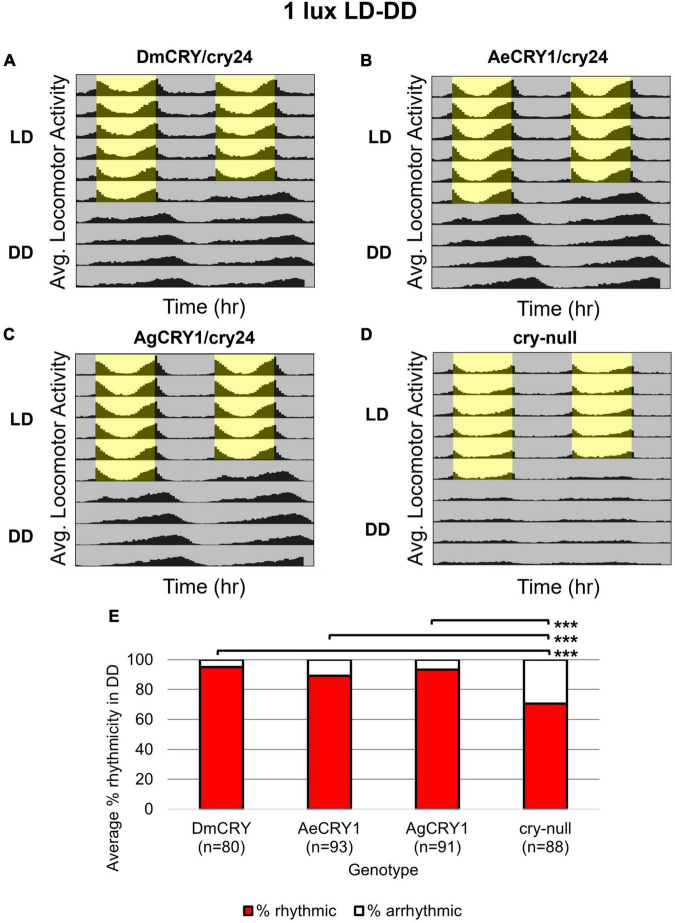
AgCRY1, AeCRY1 expressing flies and control groups maintain high rhythmicity in constant dark conditions after entrainment in low 1 lux LD white light. **(A–D)** Actogram plots containing 5 days of 12:12 h LD entrainment in 1 lux white light conditions followed by 5 days of constant dark (DD) conditions for flies expressing: **(A)** DmCRY (*n* = 80; τ_avg,DD_≈24.7, power_avg,DD_≈125.3, width_avg,DD_≈4.5), **(B)** AeCRY1 (*n* = 93; τ_avg,DD_≈24.8, power_avg,DD_≈150.6, width_avg,DD_≈5.0), **(C)** AgCRY1 (*n* = 91; τ_avg,DD_≈25.1, power_avg,DD_≈137.8, width_avg,DD_≈5.1), **(D)** *cry-null* (*n* = 88; τ_avg,DD_≈23.8, power_avg,DD_≈90.5, width_avg,DD_≈3.9). **(E)** Quantification of fly rhythmicity (red) to arrhythmicity (white) in DD. Pairwise *t*-tests were used to determine significance: *p ≤ 0.1, **p ≤ 0.05, ***p ≤ 0.01.

**FIGURE 4:**
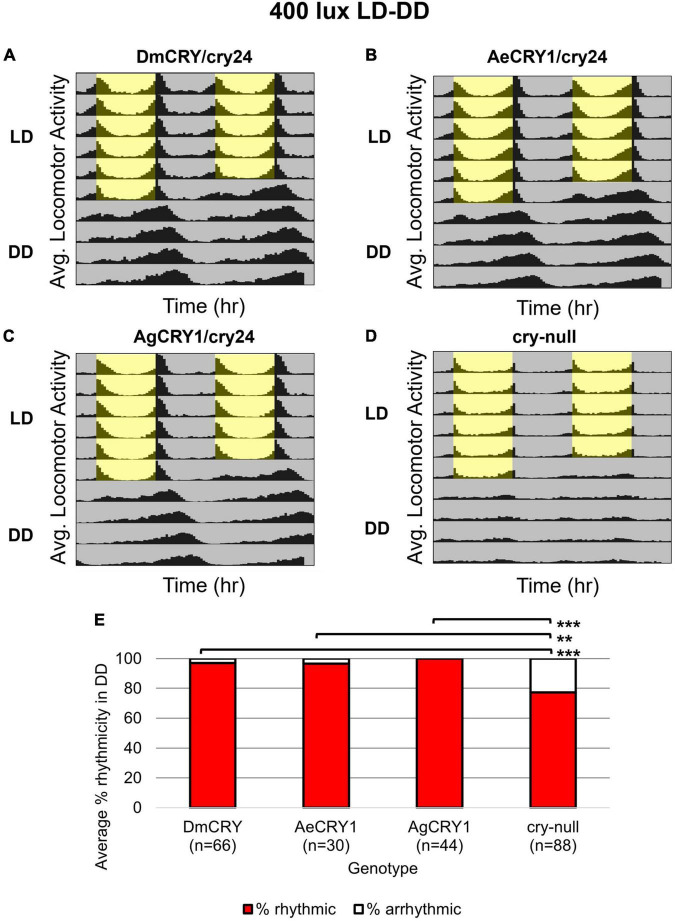
AgCRY1, AeCRY1 expressing flies and control groups maintain high rhythmicity in constant dark conditions after entrainment in moderately high 400 lux LD white light. **(A–D)** Actogram plots containing 5 days of 12:12 h LD entrainment in 400 lux white light conditions followed by 5 days of constant dark (DD) conditions for flies expressing: **(A)** DmCRY (*n* = 66; τ_avg,DD_≈24.7, power_avg,DD_≈153.2, width_avg,DD_≈4.9), **(B)** AeCRY1 (*n* = 30; τ_avg,DD_≈24.6, power_avg,DD_≈160.3, width_avg,DD_≈5.0), **(C)** AgCRY1 (*n* = 44; τ_avg,DD_≈25.4, power_avg,DD_≈137.0, width_avg,DD_≈4.9), **(D)** *cry-null* (*n* = 88; τ_avg,DD_≈23.6, power_avg,DD_≈84.5, width_avg,DD_≈3.3). **(E)** Quantification of fly rhythmicity (red) to arrhythmicity (white) in DD. Pairwise *t*-tests were used to determine significance: *p ≤ 0.1, **p ≤ 0.05, ***p ≤ 0.01.

Diurnal *Aedes aegypti* (*Ae. aegypti*) and nocturnal *Anopheles gambiae* (*An. gambiae*) mosquitoes are anthropophilic mosquitoes that occupy opposite day/night temporal niches. To determine whether heterologous CRY1 expression might disrupt the circadian clock, we compared circadian behavior in constant darkness (DD) in UAS-flies on a *cry-null* background expressing either *Drosophila* CRY (DmCRY), AeCRY1, AgCRY1 under the *crypGAL4-24* (drives expression in all cells that ordinarily express CRY, Zhao et al., 2003) vs. negative control *cry-null* flies using two white light intensities of 1 and 400 lux. The expression of AgCRY1 is not sufficient to confer nocturnal activity at either 1 or 400 lux white light (Figures 3, 4) in *Drosophila*, in contrast to the robust nocturnal behavior seen in Anopheles mosquitoes (Baik et al., 2020). For low-intensity 1 lux LD entrainment, there are no significant differences in % rhythmicity between DmCRY, AeCRY1, and AgCRY1 expressing flies (Figure 3). In contrast, *cry-null* flies show significantly less % rhythmicity relative to DmCRY, AeCRY1, and AgCRY1 expressing flies (Figure 3). Similarly, at the higher-intensity 400 lux LD entrainment, there are no significant differences in % rhythmicity between DmCRY, AeCRY1, and AgCRY1 expressing flies, while again *cry-null* flies show significantly less % rhythmicity relative to DmCRY, AeCRY1, and AgCRY1 expressing flies (Figure 4). Thus, AgCRY1 nor AeCRY1 expression disrupts the circadian clock in *Drosophila*. Further analysis shows that AgCRY1 expressing flies show significantly longer period length (tau, τ) in constant darkness compared with DmCRY, AeCRY1, and *cry-null* following 1 and 400 lux light entrainment (Supplementary Figure 1) and that *cry-null* flies show significantly shorter period length than DmCRY, AeCRY1, and AgCRY1 expressing flies following 1 and 400 lux light entrainment (Supplementary Figure 1). Further, AgCRY1 expressing flies show significantly less morning anticipatory behavior and significantly greater evening anticipatory behavior compared with DmCRY, AeCRY1, and *cry-null* during 1 and 400 lux light entrainment (Supplementary Figure 2). In an earlier paper, we also found that circadian clock function measured by free running behavior in constant darkness and morning anticipatory behavior are not well correlated (Sheeba et al., 2010). Previous work from the Helfrich-Forster group concluded that eye photoreceptor inputs are primarily responsible for modulating morning anticipation in the absence of a functional circadian clock (Schlichting et al., 2015). However, the present results suggest that Cryptochromes may also modulate morning and evening anticipation, and perhaps this is not surprising that Cryptochromes from opposing temporal niches for diurnal vs. nocturnal animals might drive differences in anticipatory behavior.

Upon photoactivation, DmCRY resets the circadian molecular clock by binding with the clock protein TIMELESS (TIM) and setting it for degradation (Emery et al., 1998; Stanewsky et al., 1998; Koh et al., 2006). The circadian clock cycles in anti-phase fashion comparing diurnal *Aedes* mosquitoes (PER levels in the s-LNv peak at ZT23) vs. nocturnal *Anopheles* mosquitoes (PER levels in the s-LNv peak at ZT11, Baik et al., 2020). To determine if diurnal AeCRY1 or nocturnal AgCRY1 is sufficient to set the circadian clock to its peak timing of TIM protein expression, transgenic flies were entrained for at least 3 days of 12:12 h Light: Dark (LD) and immunocytochemistry experiments were used to measure TIM levels at time points ZT5, ZT11, ZT17, and ZT23. Fluorescent TIM signals were quantified in the ventral lateral neuronal subgroup (LNvs) and showed peak signal at ZT23 and the lowest signals at ZT5 and ZT11 for control DmCRY, AeCRY1, and AgCRY1 expressing flies (Figures 1A–C). Negative control *cry-null* flies show a similar TIM expression pattern in the LNvs (Figure 1D). Fluorescent measurements of TIM signal during ZT17 are significantly different and are more than twofold greater in flies expressing AeCRY1 than AgCRY1, suggesting diurnal AeCRY1 is less light sensitive than nocturnal AgCRY1. However, TIM signal at ZT5, ZT11, and ZT23 does not differ between AeCRY1 and AgCRY1 flies (summary of TIM measurements, Figure 1E). Transgenic expression of mosquito CRY1 in flies also includes N-terminal fusion of eGFP for protein expression verification. DmCRY expression measured by eGFP signal shows low expression during ZT5 and ZT11 with peak expression during ZT23 (Figure 2A). AeCRY1 expression is markedly higher than DmCRY, but exhibits a similar cycling pattern with ZT5 and ZT11 showing the lowest protein levels, and ZT17 and ZT23 showing the highest protein levels (Figure 2B). AgCRY1 protein expression is consistently high during all time points (Figure 2C), but the levels are within an order of magnitude compared with DmCRY and AeCRY1 protein expression levels (summary of CRY-GFP measurements, Figure 2D). In summary, AeCRY1 and AgCRY1 expression in flies does not disrupt the circadian clock nor alter the timing of the TIM expression peak. Between genotype differences in absolute protein levels may be due to codon usage or differences in protein stability of different CRY proteins.

### *Anopheles gambiae* and *Aedes aegypti* mediate blue-light-evoked increases in electrophysiological action potential firing frequency

*Drosophila* ventral lateral neurons are circadian/arousal neurons that drive CRY-dependent acute electrophysiological light responses (Holmes et al., 2007; Sheeba et al., 2008b; Fogle et al., 2011, 2015; Giachello et al., 2016; Baik et al., 2017, 2019a; Hong et al., 2018; Au et al., 2022). We expressed AeCRY1, AgCRY1, and control DmCRY in *cry-null* genetic background flies with the UAS/GAL4 expression system, then measured the light on/light off ratio of action potential firing frequency in response to 200 μW/cm^2^ 450 nm blue-light from whole-cell patch-clamp recordings of l-LNvs in transgenic flies. For these experiments, we used the *crypGAL4-24* driver line that drives expression in all CRY neurons (Zhao et al., 2003).

**FIGURE 5:**
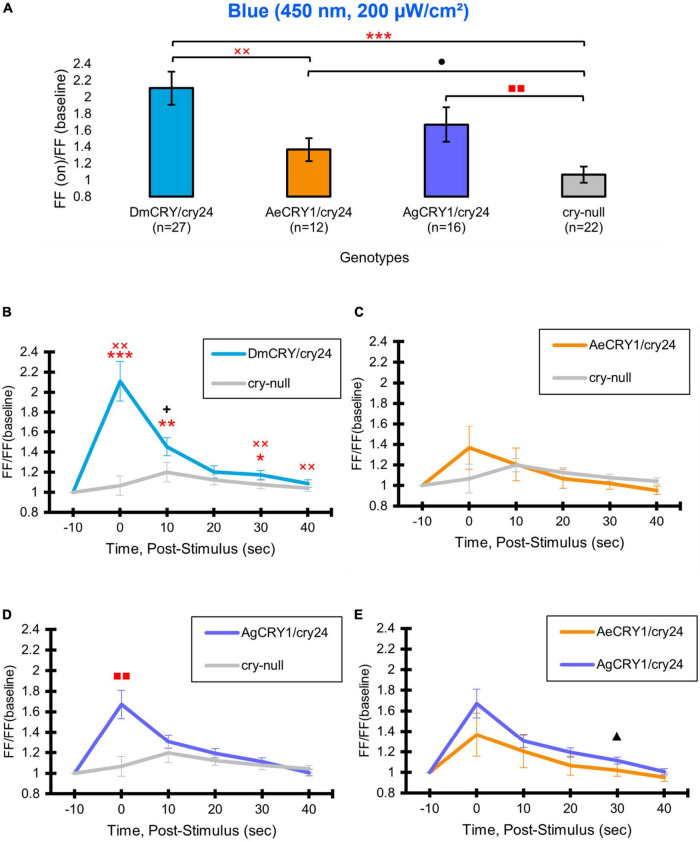
AeCRY1 and AgCRY1 mediate electrophysiological responses to blue-light. Light-evoked **(A)** FF ratio comparison of blue-light (450 nm, 200 μW/cm^2^) excited l-LNvs expressing: DmCRY (blue, *n* = 27) and negative control cry-null (gray, *n* = 22), AeCRY1 (orange, *n* = 12), and AgCRY1 (purple, *n* = 16). Light-evoked **(B–E)** post-stimulus FF comparison of blue-light (450 nm, 200 μW/cm^2^) excited l-LNvs expressing: DmCRY (blue, *n* = 27) and negative control cry-null (gray, *n* = 22), AeCRY1 (orange, *n* = 12), and AgCRY1 (purple, *n* = 16). Traces represent the average last 60 s of each recording for **(B)** DmCRY vs. cry-null, **(C)** AeCRY1 vs. cry-null, **(D)** AgCRY1 vs. cry-null, and **(E)** AeCRY1 vs. AgCRY1. Black **+** indicates two-sample *t*-test *p* ≤ 0.05 between AgCRY1/cry24 and DmCRY/cry24. Black ▲ indicates two-sample *t*-test *p* ≤ 0.05 between AgCRY1/cry24 and AeCRY1/cry24. Black • indicates two-sample *t*-test *p* ≤ 0.05 between AeCRY1/cry24 and cry-null. Red ^
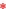
^ indicates FDR adjusted *p* ≤ 0.1 between DmCRY/cry24 and cry-null. Red 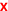 indicates *p* ≤ 0.1 between AeCRY1/cry24 and DmCRY/cry24. Red 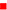 indicates FDR adjusted *p* ≤ 0.1 between AgCRY1/cry24 and cry-null. Data are represented as mean ± SEM. For black significance symbols: One symbol; *p* ≤ 0.05, two symbols; *p* ≤ 0.005, three symbols; *p* ≤ 0.001. For red significance symbols: One symbol; *p* ≤ 0.1, two symbols; *p* ≤ 0.05, three symbols; *p* ≤ 0.01.

Positive control DmCRY expression driven by the *crypGAL4-24* line mediates robust and significant increases in action potential firing frequency (FF) in the l-LNvs in response to 200 μW/cm^2^ blue-light (450 nm) relative to *cry-null* negative controls (Figure 5A, blue column vs. gray column) and mediates significant sustained increases in firing frequency in response to blue-light (Figure 5A). AeCRY1 driven by the *crypGAL4-24* line also shows significant increases in FF in the l-LNvs in response to 200 μW/cm^2^ blue-light relative to *cry-null* negative controls (Figure 5A, orange column vs. gray column). However, after adjusting for false discovery rate (FDR), there is no significance difference observed between these two groups. This is unlike AgCRY1 driven by the *crypGAL4-24* line, which shows robust and significant increases in FF in the l-LNvs in response to 200 μW/cm^2^ blue-light relative to *cry-null* negative controls (Figure 5, purple column vs. gray column) even after adjusting for FDR, suggesting a greater blue light response for AgCRY1 compared to AeCRY1. Further, the AgCRY1 blue-light FF response does not significantly differ from the DmCRY blue-light FF response (purple column vs. blue column, Figure 5A). Comparing the 200 μW/cm^2^ blue-light-evoked FF ratio during stimulus and subsequent 10 s bins post-stimulus up to 40 s, AgCRY1 FF is significantly greater than AeCRY1 FF 30 s post-stimulus (Figure 5E), but again, does not show significance after FDR adjustment. The positive control DmCRY FF is significantly greater than the *cry-null* negative control FF during stimulus and at the 10 and 30 s bins (Figure 5B).

**FIGURE 6:**
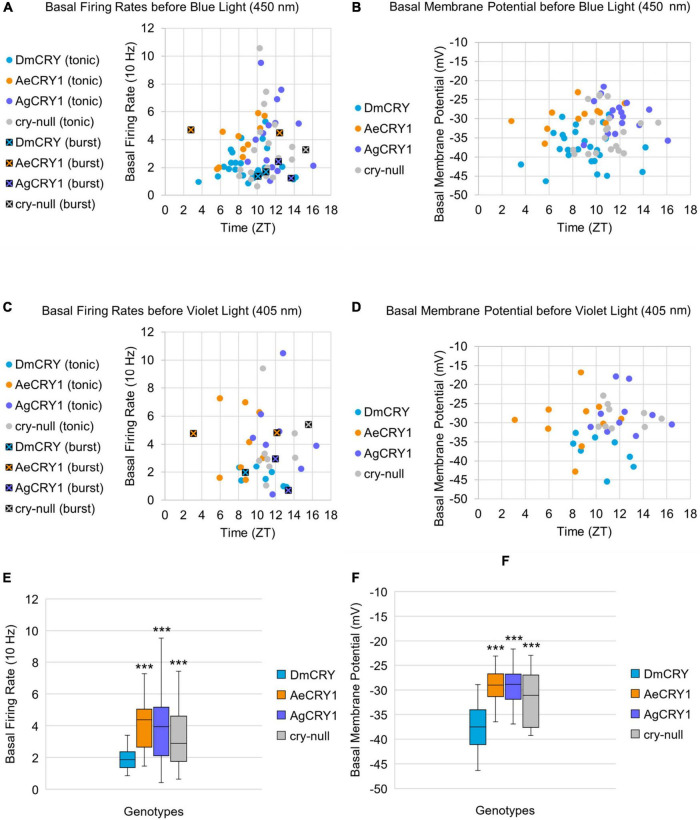
Basal firing rate and membrane potential are higher in all groups compared to the control DmCRY group and neither parameter exhibit time-of-day dependent effects. **(A)** Average basal firing rates and **(B)** average basal membrane potential before blue light stimulus plotted against the relative time-of-day of the recording for DmCRY (blue, *n* = 27), AeCRY1 (orange, *n* = 12), AgCRY1 (purple, *n* = 16), and *cry-null* (gray, *n* = 21). **(C)** Average basal firing rates and **(D)** average basal membrane potential before violet light stimulus plotted against the relative time-of-day of the recording for DmCRY (*n* = 8), AeCRY1 (*n* = 10), AgCRY1 (*n* = 10), and cry-null (*n* = 9). **(A,C)** Recordings that exhibit burst firing are denoted by a black square and cross for each respective genotype’s color. **(E,F)** Box-and-whisker plot summary of the average **(E)** basal firing rate and **(F)** basal membrane potential for DmCRY [(*n* = 35) total, n (ZT0–12) = 30; n (ZT12–16) = 5], AeCRY1 [(*n* = 22) total, n (ZT0–12) = 20; n (ZT12–16) = 2], AgCRY1 [(*n* = 26) total, n (ZT0–12) = 14; n (ZT12–16) = 12], and *cry-null* [(*n* = 30) total, n (ZT0–12) = 22; n (ZT12–16) = 8]. Median values are denoted by a solid black line within each box of the plot. Black * indicates FDR adjusted two-sample *t*-test *p* ≤ 0.01 vs. DmCRY/cry24. Data are represented as a range of means in a sample set ± maximum and minimum values within the set. One significance symbol; *p* ≤ 0.1, two significance symbols; *p* ≤ 0.05, three significance symbols; *p* ≤ 0.01.

Previous work shows that light activated CRY mediates changes in membrane potential through the voltage gated potassium channel beta subunit and modulation of potassium channels (Fogle et al., 2011, 2015; Giachello et al., 2016; Baik et al., 2017, 2018, 2019a; Hong et al., 2018; Tabuchi et al., 2021). To determine whether mosquito CRY expression alters LNv basal electrophysiological processes, we plotted basal l-LNv firing rates, basal resting membrane potential values and firing mode (tonic vs. burst firing) across the time of day of the recordings (Figure 6). The range of l-LNv firing rates and the average resting membrane potentials from the present set of whole-cell patch-clamp recordings for DmCRY expressing neurons are similar to previously reported values around –40 mV (the mean is −37 mV, Figures 6B,D,F). Basal firing rates and resting membrane potentials for DmCRY expressing flies are significantly lower than *cry-null*, AeCRY1 and AgCRY1 expressing flies (Figures 6E,F). The majority of the l-LNv recordings are from neurons during the day between ZT6-ZT12 and include a few recordings for the first few hours of night up until ZT16. None of the genotypes shows clear time of day differences in basal firing rate or membrane resting potential. However, these experiments were not designed to test time of day distributions as the present data cluster during midday. There are relatively few nighttime recordings and recordings from early morning and late night are not represented. Previous publications designed to test this question, including several of our own, show firing rates trending high at the beginning of day that tend to decrease at night (Cao and Nitabach, 2008; Sheeba et al., 2008b,^2010^; Flourakis et al., 2015; Smith et al., 2019). Consistent with most earlier publications, we observe predominantly tonic action potential firing in l-LNv recordings (Holmes et al., 2007; Cao and Nitabach, 2008; Sheeba et al., 2008b,^2010^; McCarthy et al., 2011; Seluzicki et al., 2014; Flourakis and Allada, 2015; Flourakis et al., 2015; Fogle et al., 2015; Buhl et al., 2016, 2019; Baik et al., 2017, 2019a; Li et al., 2018; Smith et al., 2019; Au et al., 2022). Burst firing as the predominant firing mode in l-LNv has been reported by another group (Muraro and Ceriani, 2015; Fernandez-Chiappe et al., 2021), however, they do not systematically address firing mode as a function of time of day.

**FIGURE 7:**
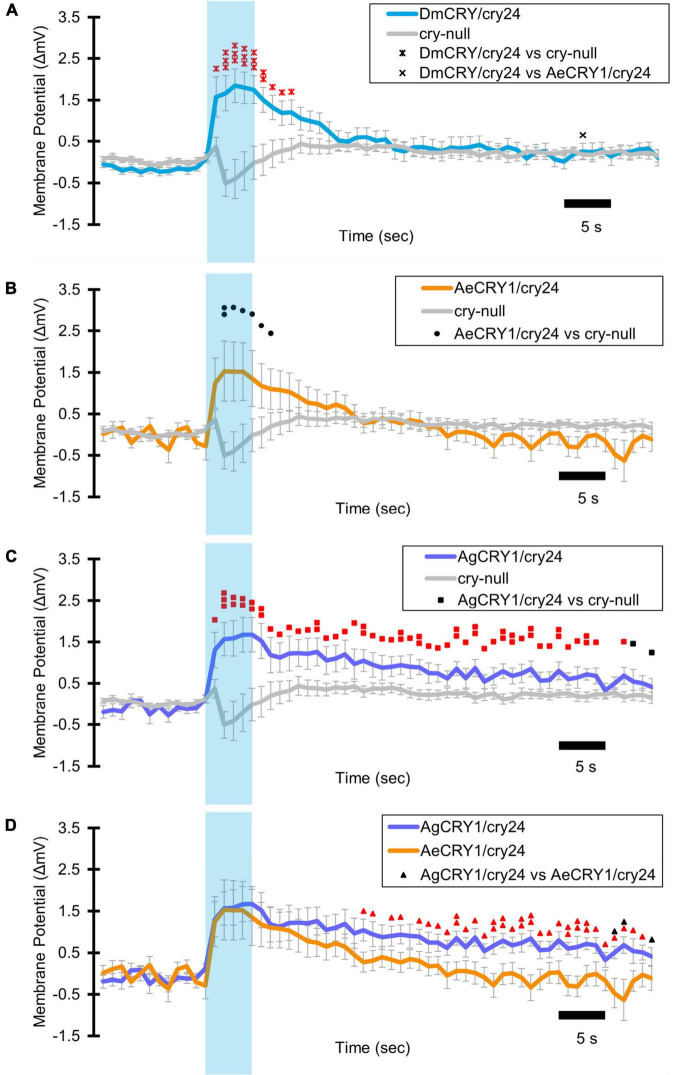
AgCRY1 mediate significantly greater and sustained membrane depolarization in responses to blue-light compared to AeCRY1. Light-evoked **(A–D)** membrane potential comparison of blue-light (450 nm, 200 μW/cm^2^) excited l-LNvs expressing: DmCRY (blue, *n* = 27) and negative control *cry-null* (gray, *n* = 22), AeCRY1 (orange, *n* = 12), and AgCRY1 (purple, *n* = 16). Blue bar on membrane potential plots indicates the timing of the 5 s of blue-light stimuli and black scale-bar indicates 5 s. Traces represent the average last 60 s of each recording for **(A)** DmCRY vs. *cry-null*, **(B)** AeCRY1 vs. *cry-null*, **(C)** AgCRY1 vs. *cry-null*, and **(D)** AeCRY1 vs. AgCRY1. Black • indicates two-sample *t*-test *p* ≤ 0.05 between AeCRY1/cry24 and *cry-null*. Black ▲ indicates two-sample *t*-test *p* ≤ 0.05 between AgCRY1/cry24 and AeCRY1/cry24. Black **x** indicates two-sample *t*-test *p* ≤ 0.05 between DmCRY/cry24 and AeCRY1/cry24. Black ◼ indicates two-sample *t*-test *p* ≤ 0.05 between AgCRY1/cry24 and *cry-null*. Black ▲ indicates two-sample *t*-test *p* ≤ 0.05 between AgCRY1/cry24 and AeCRY1/cry24. Red ^
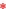
^ indicates FDR adjusted *p* ≤ 0.1 between DmCRY/cry24 and *cry-null*. Red 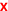 indicates *p* ≤ 0.1 between AeCRY1/cry24 and DmCRY/cry24. Red 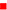 indicates FDR adjusted *p* ≤ 0.1 between AgCRY1/cry24 and *cry-null*. Red 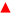 indicates FDR adjusted *p* ≤ 0.1 between AgCRY1/cry24 and AeCRY1/cry24. Data are represented as mean ± SEM. For black significance symbols: One symbol; *p* ≤ 0.05, two symbols; *p* ≤ 0.005, three symbols; *p* ≤ 0.001. For colored significance symbols: One symbol; *p* ≤ 0.1, two symbols; *p* ≤ 0.05, three symbols; *p* ≤ 0.01.

**FIGURE 8:**
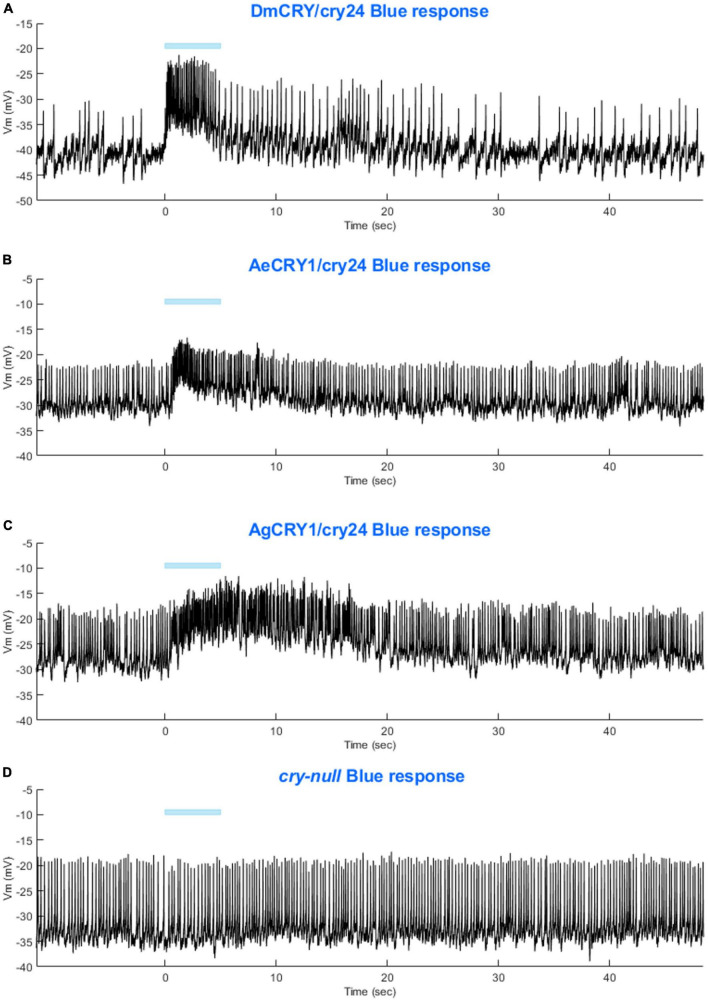
Representative voltage traces of l-LNvs electrophysiological responses to blue-light stimuli for all genotypes. Representative voltage traces of the last 60 s of a patch-clamp recording of l-LNvs subjected to 5 s of blue-light stimuli for **(A)** DmCRY/cry24, **(B)** AeCRY1/cry24, **(C)** AgCRY1/cry24, and **(D)** *cry-null* flies. Blue bar indicates 5 s of 200 μW/cm^2^ blue-light stimulus.

Light-evoked averaged potentials are more kinetically reliable than light onset and CRY mediated action potential firing (Fogle et al., 2011; Baik et al., 2019a; Au et al., 2022). The blue-light-evoked response of DmCRY relative to the *cry-null* negative control shows strong depolarization then a slowly tapering sustained response over the 10 s following light stimulus offset (Figure 7A) with a qualitatively similar response recorded from neurons expressing AeCRY1 relative to the *cry-null* negative control (Figure 7B). In contrast, the blue-light-evoked response of neurons expressing AgCRY1 relative to the *cry-null* negative control show sustained significant depolarization during lights on, followed by a very long sustained depolarization response that lasts tens of seconds (Figure 7C). The blue-light response of AgCRY1 relative to AeCRY1 exhibits a significantly longer and more sustained membrane depolarization event lasting for tens of seconds evoked by a 5 s pulse of 200 μW/cm^2^ blue-light relative to the shorter-lasting AeCRY1 evoked blue-light potential (Figure 7D). The significantly higher AgCRY1 blue-light-evoked depolarization for most of the duration of the evoked potential occurs after approximately 15 s post-stimulus relative to AeCRY1 (Figure 7D). These results, particularly the similar duration of the evoked potential blue-light response between AeCRY1 and DmCRY, suggest no direct relationship between CRY expression levels (Figure 3) and the magnitude of the physiological light response (Figures 2, 5, 6), confirming earlier findings concerning this (Baik et al., 2017, 2019a,b; Au et al., 2022). The AgCRY1 blue-light-evoked potential is significantly greater than that for the *cry-null* negative control for almost the entire duration up to 40 s from the stimulus onset (Figure 7C), while the much weaker AeCRY1 evoked potential is only significantly higher than the *cry-null* negative control for the first few seconds following stimulus onset (Figure 7B), but after FDR adjustment, it does not show significant differences. AgCRY1 confers a more sustained light response than DmCRY (Figures 7A,C). Representative voltage traces showing light-evoked depolarization and increased action potential firing frequency in patch-clamp recordings of l-LNvs during the 5 s of blue-light stimuli and 60 s post-light stimulus for positive control DmCRY/cry24, AeCRY1/cry24, AgCRY1/cry24, and negative control *cry-null* flies are shown in Figure 8, where the blue bar indicates 5 s of 200 μW/cm^2^ 450 nm blue-light stimulus.

**FIGURE 9:**
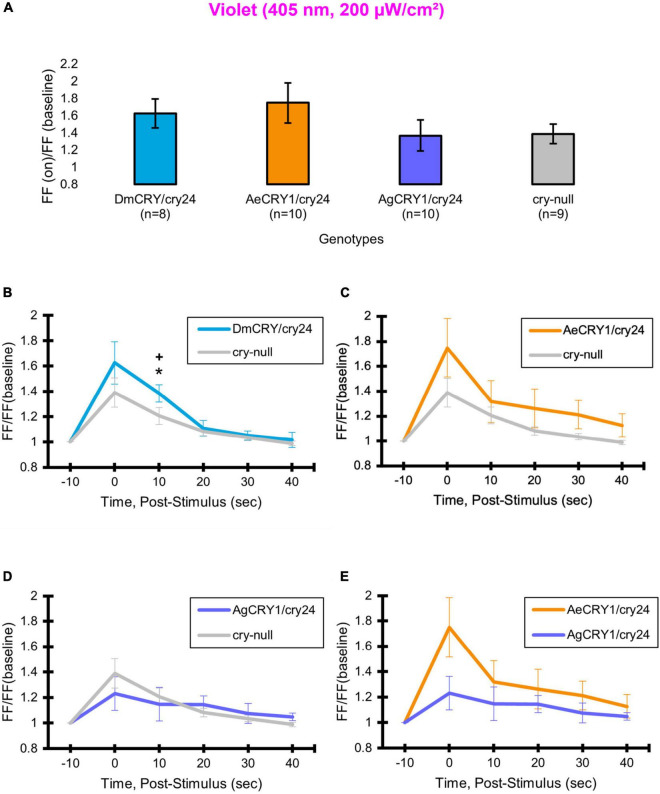
AeCRY1 and AgCRY1 FF ratios shows weak responses to violet-light. Light-evoked **(A)** FF ratio comparison of violet-light (405 nm, 200 μW/cm^2^) excited l-LNvs expressing: DmCRY (blue, *n* = 8) and negative control cry-null (gray, *n* = 9), AeCRY1 (orange, *n* = 10), and AgCRY1 (purple, *n* = 10). Light-evoked **(B–E)** post-stimulus FF comparison of violet-light (405 nm, 200 μW/cm^2^) excited l-LNvs expressing: DmCRY (blue, *n* = 8) and negative control cry-null (gray, *n* = 9), AeCRY1 (orange, *n* = 10), and AgCRY1 (purple, *n* = 10). Traces represent the average last 60 s of each recording for **(B)** DmCRY vs. cry-null, **(C)** AeCRY1 vs. cry-null, **(D)** AgCRY1 vs. cry-null, and **(E)** AeCRY1 vs. AgCRY1. Black * indicates two-sample *t*-test *p* ≤ 0.05 between DmCRY/cry24 and cry-null. Black + indicates two-sample *t*-test *p* ≤ 0.05 between AgCRY1/cry24 and DmCRY/cry24. Data are represented as mean ± SEM. For black significance symbols: One symbol; *p* ≤ 0.05, two symbols; *p* ≤ 0.005, three symbols; *p* ≤ 0.001.

**FIGURE 10:**
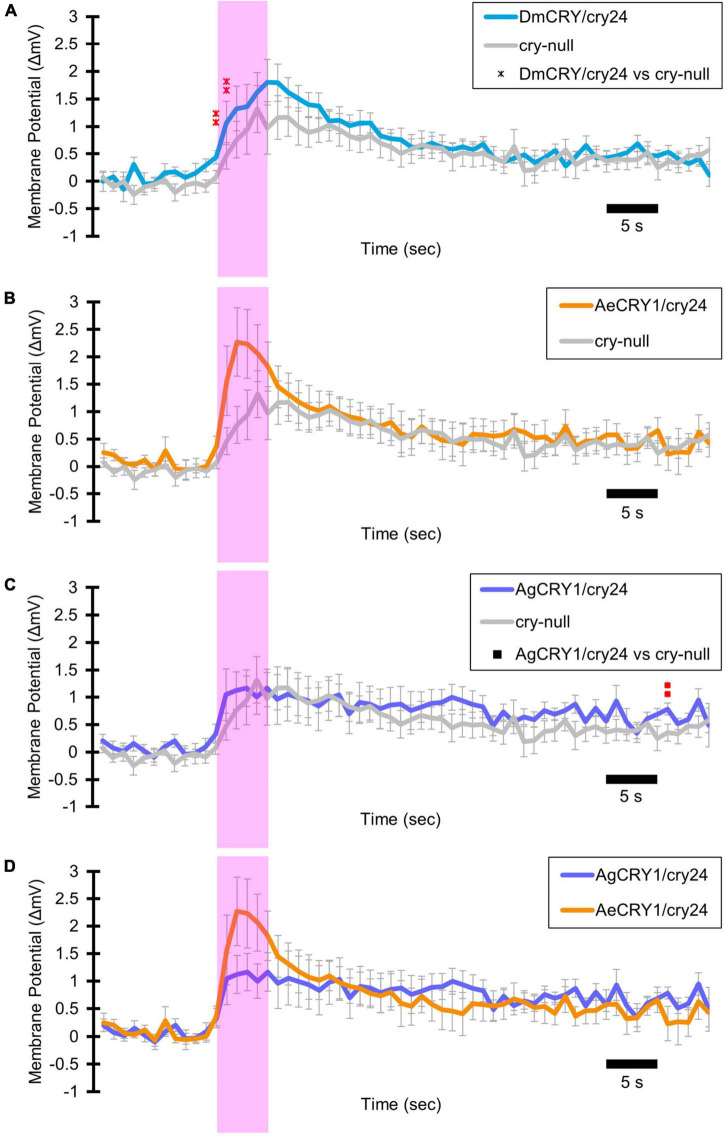
AeCRY1 and AgCRY1 RMP mediate weak membrane-evoked responses to violet-light. Light-evoked **(A–D)** membrane potential comparison of violet-light (405 nm, 200 μW/cm^2^) excited l-LNvs expressing: DmCRY (blue, *n* = 8) and negative control cry-null (gray, *n* = 9), AeCRY1 (orange, *n* = 10), and AgCRY1 (purple, *n* = 10). Violet bar on membrane potential plots indicates the timing of the 5 s of violet-light stimuli and black scale-bar indicates 5 s. Traces represent the average last 60 s of each recording for **(A)** DmCRY vs. cry-null, **(B)** AeCRY1 vs. cry-null, **(C)** AgCRY1 vs. cry-null, and **(D)** AeCRY1 vs. AgCRY1. Red ^
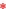
^ indicates FDR adjusted *p* ≤ 0.1 between DmCRY/cry24 and cry-null. Red 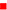 indicates FDR adjusted *p* ≤ 0.1 between AgCRY1/cry24 and cry-null. Data are represented as mean ± SEM. For red significance symbols: One symbol; *p* ≤ 0.1, two symbols; *p* ≤ 0.05, three symbols; *p* ≤ 0.01.

**FIGURE 11:**
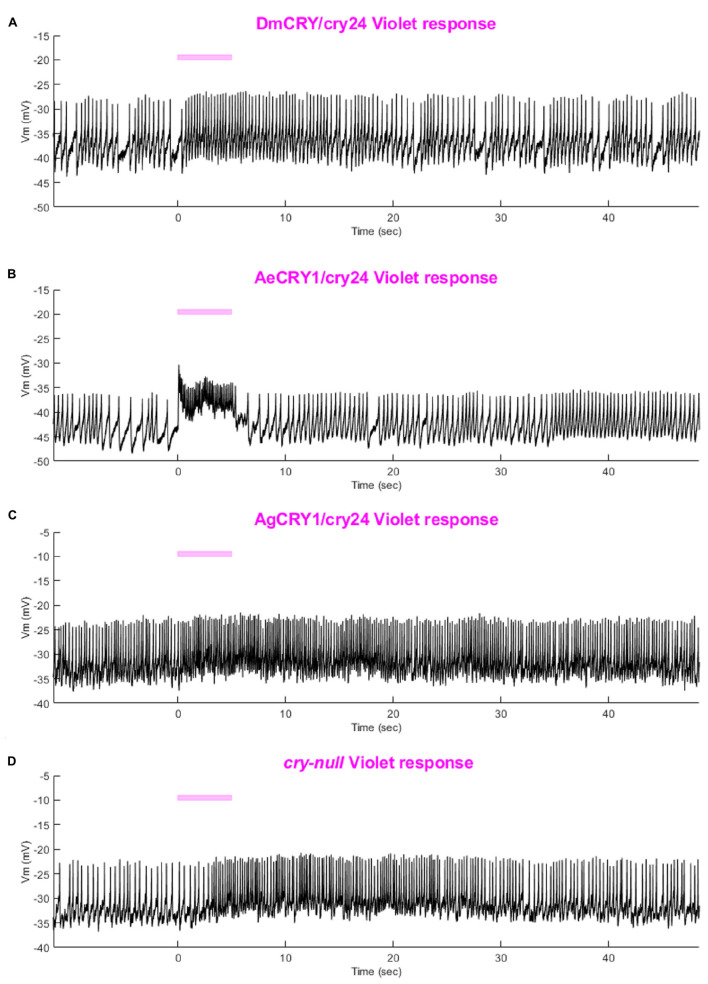
Representative voltage traces of l-LNvs electrophysiological response to violet light stimuli for all genotypes. Representative voltage traces of the last 60 s of a patch-clamp recording of l-LNvs subjected to 5 s of violet-light stimuli for **(A)** DmCRY/cry24, **(B)** AeCRY1/cry24, **(C)** AgCRY1/cry24, and **(D)** *cry-null* flies. Violet bar indicates 5 s of 200 μW/cm^2^ violet-light stimulus.

As expected, there are no significant differences in light-evoked FF between all four CRY genotypes in response to 200 μW/cm^2^ violet-light (405 nm) (Figure 9A), as there is a trough of the CRY action spectra around 405 nm and Rh7 and other opsin photoreceptors are activated in this range of the color spectra (Ni et al., 2017; Sakai et al., 2017; Baik et al., 2019b). The depolarization magnitude and duration of DmCRY, AeCRY1, AgCRY1, and negative control *cry-null* responses to violet-light are similar and indistinguishable from *cry-null* and are at a lower magnitude of FF ratio and depolarization magnitude and duration relative to intensity matched blue-light stimuli (compare Figure 9 vs. Figure 5). The violet-light-evoked increases in l-LNv firing frequency (Figures 9B–E) and light-evoked depolarization (Figure 10) during and after the violet-light stimulus are weak and do not differ systematically between the different CRY genotypes. These results are consistent with earlier findings that CRY is not activated by violet-light and is consistent with earlier findings that Rh7 is the primary non-image forming visual violet-light photoreceptor in LNvs (Ni et al., 2017; Sakai et al., 2017; Baik et al., 2019b). Representative voltage traces showing light-evoked depolarization and increased action potential firing frequency in patch-clamp recordings of l-LNvs during the 5 s of violet-light stimuli and 60 s post-light stimulus for positive control DmCRY/cry24, AeCRY1/cry24, AgCRY1/cry24, and negative control *cry-null* flies are shown in Figure 11, where the violet bar indicates 5 s of 200 μW/cm^2^ 405 nm violet-light stimulus.

### Diurnal/nocturnal mosquito CRY1s confer species-specific and intensity-dependent behavioral attraction/avoidance responses to blue and violet-light

**FIGURE 12:**
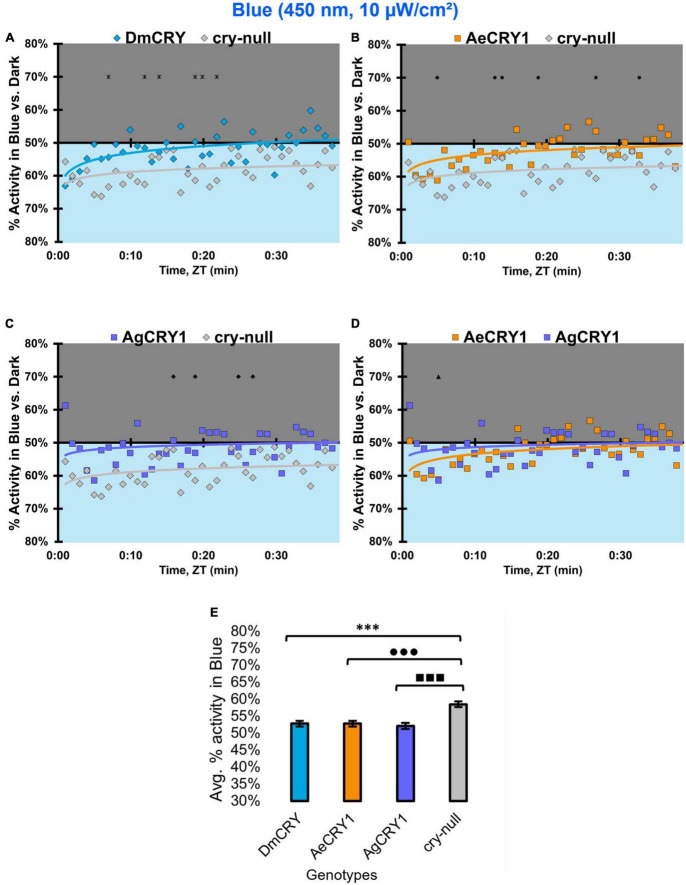
All transgenic groups exhibit little or no behavioral attraction to low-intensity blue-light. **(A–D)** Blue attraction/avoidance behavior is measured by % activity in a dark shaded environment vs. a low-intensity (10 μW/cm^2^) blue-light-exposed environments (450 nm) during the light phase of a standard 12:12 h LD cycle. Preference is calculated by percentage of activity in each environment over total activity for each time bin for **(A)** DmCRY (blue, *n* = 53) vs. *cry-null* (red, *n* = 53), **(B)** diurnal AeCRY1 (orange, *n* = 46) vs. *cry-null*, **(C)** nocturnal AgCRY1 (purple, *n* = 47) vs. *cry-null*, and **(D)** AeCRY1 vs. AgCRY1. All plots are shown from ZT0 to 30 min in 1-min bins. **(E)** Quantified mean % activity of flies in blue environment across the first 30 min for low-intensity blue-light environments. Black * indicates two-sample *t*-test *p* ≤ 0.05 between DmCRY/cry24 and *cry-null*. Black • indicates two-sample *t*-test *p* ≤ 0.05 between AeCRY1/cry24 and *cry-null*. Black ■ indicates two-sample *t*-test *p* ≤ 0.05 between AgCRY1/cry24 and *cry-null*. Black ▲ indicates two-sample *t*-test *p* ≤ 0.05 between AgCRY1/cry24 and AeCRY1/cry24. Data are represented as mean ± SEM. One significance symbol; *p* ≤ 0.05, two significance symbols; *p* ≤ 0.005, three significance symbols; *p* ≤ 0.001.

Diurnal mosquitoes are behaviorally attracted to short-wavelength light (UV, blue), while nocturnal mosquitoes behaviorally avoid short wavelength light (Baik et al., 2020). CRY1 is a strong photoreceptor candidate to drive these species-specific attraction/avoidance behavioral light responses. In our recent study Au et al. (2022) testing transgenic *Drosophila* that express diurnal AeCRY1 or nocturnal AgCRY1 in a *cry-null* genetic background, we find that AeCRY1 expressing flies show strong photo-attraction behavioral responses to a wide intensity range (1–400 μW/cm^2^) of UV (365 nm) light. In contrast, nocturnal AgCRY1 expressing flies show discernable photo-attraction behavioral responses to UV light at very low intensities (1 μW/cm^2^) but show significant photo-avoidance behavioral responses to higher UV light intensities (at 10 and 400 μW/cm^2^ of UV light). Here, we examine the role for CRY1s for conferring day- vs. night-active mosquito species-specific light choice behaviors to other wavelengths by performing blue (450 nm) and violet (405 nm) light choice behavioral assays with flies expressing DmCRY, AgCRY1, AeCRY1 under the *crypGAL4-24* promoter at low (10 μW/cm^2^) and high (400 μW/cm^2^) light intensities using an environmental light choice preference test. At low intensity (10 μW/cm^2^) 450 nm blue-light, *cry-null* flies show significantly greater attraction to blue-light relative to all CRY expressing fly groups (Figures 12A–C). Flies expressing DmCRY, AgCRY1, or AeCRY1 show weak or no behavioral attraction to low intensity blue-light (Figures 12A–D). The average% activity of flies in the blue lit environment over the first 30 min shows no significant differences between flies expressing DmCRY, AeCRY1, or AgCRY1 (Figure 12E).

**FIGURE 13:**
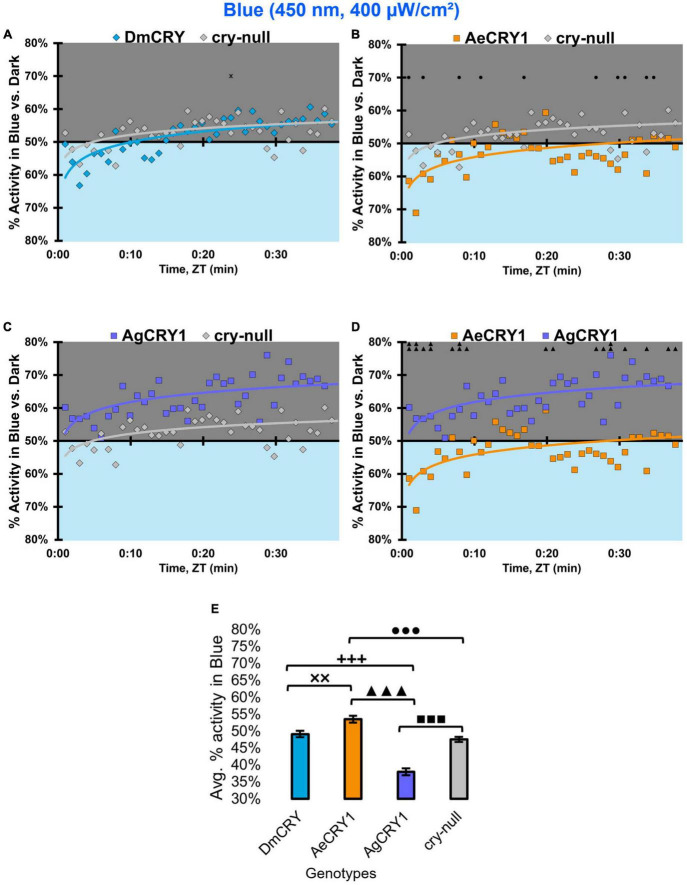
AgCRY1 flies behaviorally avoid high intensity blue-light. **(A–D)** Blue attraction/avoidance behavior is measured by % activity in a dark shaded environment vs. a high-intensity (400 μW/cm^2^) blue-light-exposed environments (450 nm) during the light phase of a standard 12:12 h LD cycle. Preference is calculated by percentage of activity in each environment over total activity for each time bin for **(A)** DmCRY (blue, *n* = 52) vs. *cry-null* (red, *n* = 51), **(B)** diurnal AeCRY1 (orange, *n* = 39) vs. *cry-null*, **(C)** nocturnal AgCRY1 (purple, *n* = 46) vs. *cry-null*, and **(D)** AeCRY1 vs. AgCRY1. All plots are shown from ZT0 to 30 min in 1-min bins. **(E)** Quantified mean % activity of flies in blue environment across the first 30 min for high-intensity blue-light environments. Black * indicates two-sample *t*-test *p* ≤ 0.05 between DmCRY/cry24 and *cry-null*. Black ◼ indicates two-sample *t*-test *p* ≤ 0.05 between AgCRY1/cry24 and *cry-null*. Black • indicates two-sample *t*-test *p* ≤ 0.05 between AeCRY1/cry24 and *cry-null*. Black ▲ indicates two-sample *t*-test *p* ≤ 0.05 between AgCRY1/cry24 and AeCRY1/cry24. Black **+** indicates two-sample *t*-test *p* ≤ 0.05 between AgCRY1/cry24 and DmCRY/cry24. Black **x** indicates two-sample *t*-test *p* ≤ 0.05 between AeCRY1/cry24 and DmCRY/cry24. Data are represented as mean ± SEM. One significance symbol; *p* ≤ 0.05, two significance symbols; *p* ≤ 0.005, three significance symbols; *p* ≤ 0.001.

In contrast, at higher intensity 400 μW/cm^2^ 450 nm blue-light, the genotypes behavioral light responses diverge: DmCRY expressing flies exhibit relatively neutral responses to the blue lit environment, showing moderate photo-attraction for 15 min of blue-light exposure, then moderate photo-avoidance to 400 μW/cm^2^ 450 nm blue-light for the next 15 min (Figure 13A). AeCRY1 expressing flies show significantly greater behavioral attraction to high intensity blue-light relative to *cry-null* and AgCRY1 expressing flies at many time points (Figures 13B,D). AgCRY1 expressing flies exhibit the greatest significant light avoidance to the high-intensity blue-light exposed environment relative to other genotypes (Figures 13A–E). This is confirmed by average% activity plots for each CRY expressing genotype showing that AeCRY1 expressing flies have significantly greater activity in higher intensity blue-light than either AgCRY1 or DmCRY, and that AgCRY1 have significantly the least amount of activity in high intensity blue-light relative to AeCRY1 or DmCRY (Figure 13E).

**FIGURE 14:**
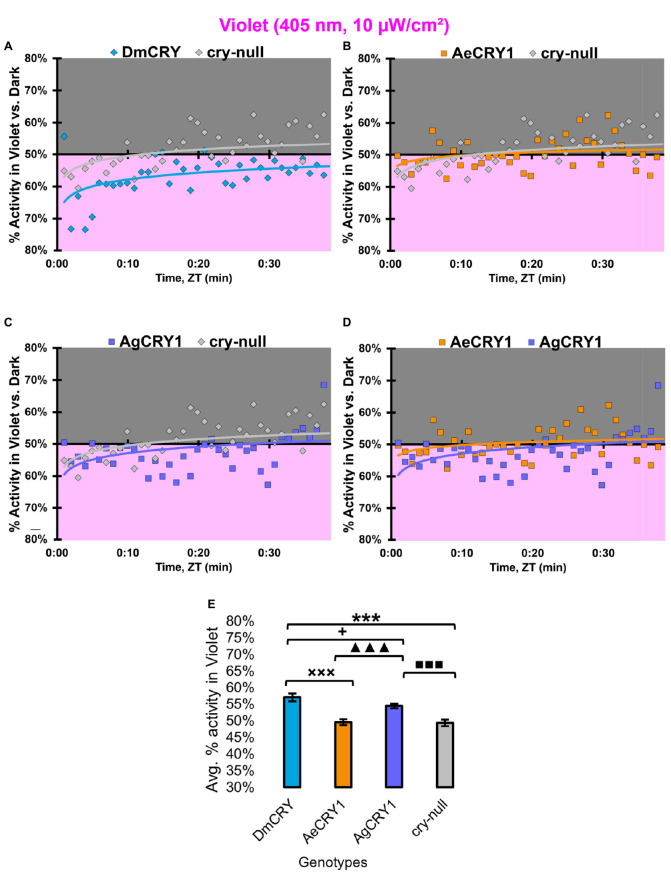
All transgenic groups exhibit weak-moderate behavioral attraction to low-intensity violet-light. **(A–D)** Violet attraction/avoidance behavior is measured by % activity in a dark shaded environment vs. a moderately low-intensity (10 μW/cm^2^) violet-light-exposed environments (405 nm) during the light phase of a standard 12:12 h LD cycle. Preference is calculated by percentage of activity in each environment over total activity for each time bin for **(A)** DmCRY (blue, *n* = 43) vs. *cry-null* (red, *n* = 42), **(B)** diurnal AeCRY1 (orange, *n* = 35) vs. *cry-null*, **(C)** nocturnal AgCRY1 (purple, *n* = 36) vs. *cry-null*, and **(D)** AeCRY1 vs. AgCRY1. All plots are shown from ZT0 to 30 min in 1-min bins. **(E)** Quantified mean % activity of flies in violet environment across the first 30 min for moderately low-intensity violet-light environments. Black * indicates two-sample *t*-test *p* ≤ 0.05 between DmCRY/cry24 and *cry-null*. Black ◼ indicates two-sample *t*-test *p* ≤ 0.05 between AgCRY1/cry24 and *cry-null*. Black **+** indicates two-sample *t*-test *p* ≤ 0.05 between AgCRY1/cry24 and DmCRY/cry24. Black **x** indicates two-sample *t*-test *p* ≤ 0.05 between AeCRY1/cry24 and DmCRY/cry24. Black ▲ indicates two-sample *t*-test *p* ≤ 0.05 between AgCRY1/cry24 and AeCRY1/cry24. Data are represented as mean ± SEM. One significance symbol; *p* ≤ 0.05, two significance symbols; *p* ≤ 0.005, three significance symbols; *p* ≤ 0.001.

**FIGURE 15:**
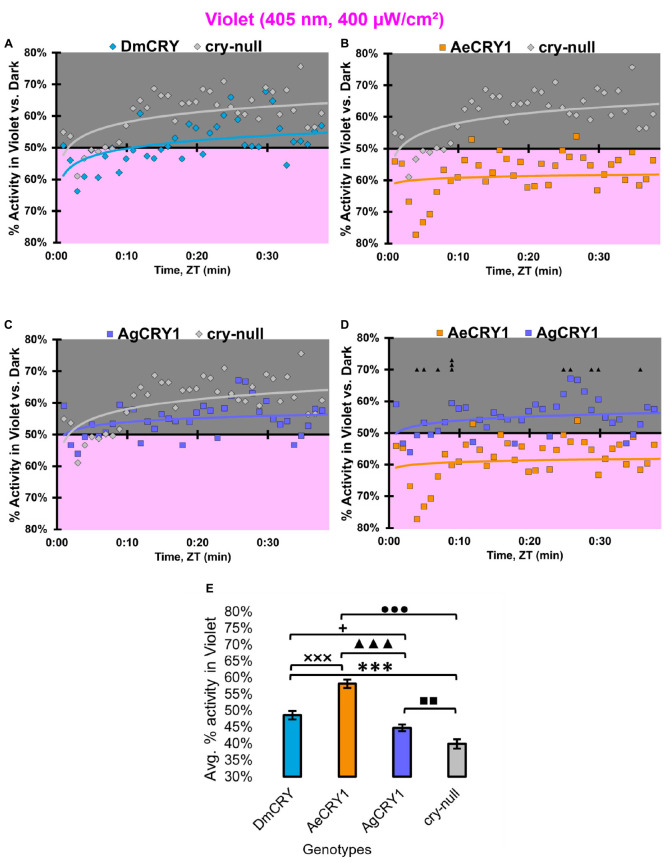
AeCRY1 flies exhibit behavioral attraction to high intensity violet-light. **(A–D)** Violet attraction/avoidance behavior is measured by % activity in a dark shaded environment vs. a high-intensity (400 μW/cm^2^) violet-light-exposed environments (405 nm) during the light phase of a standard 12:12 h LD cycle. Preference is calculated by percentage of activity in each environment over total activity for each time bin for **(A)** DmCRY (blue, *n* = 35) vs. *cry-null* (red, *n* = 40), **(B)** diurnal AeCRY1 (orange, *n* = 34) vs. *cry-null*, **(C)** nocturnal AgCRY1 (purple, *n* = 40) vs. *cry-null*, and **(D)** AeCRY1 vs. AgCRY1. All plots are shown from ZT0 to 30 min in 1-min bins. **(E)** Quantified mean % activity of flies in violet environment across the first 30 min for high-intensity violet-light environments. Black ▲ indicates two-sample *t*-test *p* ≤ 0.05 between AgCRY1/cry24 and AeCRY1/cry24. Black * indicates two-sample *t*-test *p* ≤ 0.05 between DmCRY/cry24 and *cry-null*. Black ◼ indicates two-sample *t*-test *p* ≤ 0.05 between AgCRY1/cry24 and *cry-null*. Black • indicates two-sample *t*-test *p* ≤ 0.05 between AeCRY1/cry24 and cry-null. Black **+** indicates two-sample *t*-test *p* ≤ 0.05 between AgCRY1/cry24 and DmCRY/cry24. Black x indicates two-sample *t*-test *p* ≤ 0.05 between AeCRY1/cry24 and DmCRY/cry24. Data are represented as mean ± SEM. One significance symbol; *p* ≤ 0.05, two significance symbols; *p* ≤ 0.005, three significance symbols; *p* ≤ 0.001.

At low intensity 405 nm violet-light (10 μW/cm^2^), DmCRY and AgCRY1 expressing flies both show behavioral photo-attraction to the low intensity violet lit environment (Figures 14A,C,D), while *cry-null* and AeCRY1 expressing flies show less behavioral photo-attraction to the violet lit environment (Figures 14B,D). The average% activity plots for each CRY expressing genotype shows AeCRY1 expressing flies show significantly the least behavioral activity in low intensity violet-light while DmCRY expressing flies show significantly the most behavioral activity in low intensity violet-light (Figure 14E). Control *cry-null* and DmCRY expressing flies both behaviorally avoid high intensity violet-light (400 μW/cm^2^, Figure 15A), except during the first 10 min of violet-light exposure for DmCRY expressing flies. The behavioral responses to high intensity violet-light are divergent between AgCRY1 and AeCRY1 expressing flies: AgCRY1 expressing flies behaviorally avoid high intensity violet-light while AeCRY1 expressing flies are behaviorally attracted to high intensity violet-light, consistent with the previously reported general attraction of *Ae. aegypti* mosquitoes to all visible light wavelengths (Figures 15A–E, see also Baik et al., 2020). The average% activity plots for each CRY expressing genotype shows that AeCRY1 expressing flies show significantly the greatest behavioral activity in high intensity violet-light while AgCRY1 expressing flies show significantly the least behavioral activity in high intensity violet-light (Figure 15E). Taken together for responses to varying intensities of violet-light, these complex behavioral effects may be due either to direct effects through mosquito CRY proteins or possibly due to unknown CRY interactions with the major violet-light sensor Rh7 that co-expresses in the LNv subgroups to mediate multi-photoreceptor inputs for light attraction/avoidance behavioral responses (Ni et al., 2017; Baik et al., 2018, 2019b), or image forming photoreception in the eyes Altogether, these results indicate the blue and violet-light intensity-dependent light attraction/avoidance behaviors significantly diverge between AeCRY1 and AgCRY1 expressing flies and that these behavioral results are consistent with the distinct diurnal and nocturnal mosquito attraction/avoidance responses to short-wavelength light. Taken together, the data provides further support to our conclusions that CRY photoreceptors mediate species-specific physiological and behavioral light responses (Baik et al., 2020; Au et al., 2022).

## Discussion

This work was motivated by our recent findings that diurnal *Ae. aegypti* mosquitoes and nocturnal *An. coluzzii* (gambiae sub-family) mosquitoes exhibit very different attraction/avoidance behavioral responses to different light spectra that vary by time of day; and that these light driven behaviors are modulated by CRY in mosquitoes (Baik et al., 2020). We considered multiple hypotheses that might account for the distinct physiological light responses of diurnal and nocturnal mosquitoes and tested the simplest and most tractable hypothesis: informed by earlier work showing that *Drosophila* CRY codes for light avoidance responses to high intensity short wavelength light (Baik et al., 2017, 2018, 2019b), we tested the hypothesis that there are species-specific differences in the CRY light responses between *Ae. aegypti* and *An. gambiae* -family mosquitoes, predicting that nocturnal *An. gambiae* CRY1 exhibits stronger electrophysiological and behavioral responses to blue-light than *Ae. aegypti* CRY1. For the present work, the comparison between blue and violet-light responses is logically dictated by the relative spectral absorbance profiles of two non-imaging forming photoreceptors, CRY and Rh7 (Ni et al., 2017, p. 7; Sakai et al., 2017). Rh7 exhibits a broad absorption spectrum that peaks in the violet range while the base state of CRY shows a trough in the violet range of the spectra.

We recently published a related study comparing the effects of expressing the light sensitive CRYs from *Ae. aegypti* (AeCRY1), *An. gambiae* (AgCRY1), and *Drosophila melanogaster* (DmCRY, a positive control in a *cry-null Drosophila melanogaster* genetic background) in Au et al. (2022). While DmCRY is included as a positive control for the physiological assays, we acknowledge that DmCRY is a native protein in flies while mosquito CRYs are heterologously expressed. AeCRY1 is much less light sensitive than either AgCRY1 or DmCRY as shown by numerous physiological assays including partial behavioral rhythmicity seen in AeCRY1 expressing flies following constant light exposure (Au et al., 2022) and herein. Remarkably, expression of nocturnal AgCRY1 confers low survival to constant white light exposure as does expression of AeCRY1 to a much lesser extent, which may contribute to enforcing species-specific time-of-day behavioral activity. In that study, we show that AgCRY1 mediates significantly stronger electrophysiological cell autonomous responses to 365 nm ultraviolet (UV) light relative to AeCRY1 (Au et al., 2022). Further, AgCRY1 expression mediates electrophysiological and behavioral sensitivity to 635 nm red-light while AeCRY1 does not, consistent with species-specific mosquito red-light responses (Baik et al., 2020; Au et al., 2022). AgCRY1 and DmCRY mediate intensity-dependent avoidance behavior to UV light at different light intensity thresholds, while AeCRY1 does not, thus mimicking mosquito and fly behaviors (Au et al., 2022). These findings along with the present findings showing physiological responses to blue and violet-light collectively highlight CRY as a key non-image forming visual photoreceptor that mediates physiological and behavioral light-responses in a species-specific fashion.

Several mechanisms mediate inter-protein signaling following CRY light activation. For CRY mediated clock resetting in *Drosophila*, there is clear evidence that light activation leads to conformational changes in the CRY c-terminal tail that signal to downstream proteins (Busza et al., 2004; Dissel et al., 2004; Ozturk et al., 2011). However, CRY mediated light-evoked increases in action potential firing rate is still observed in flies that express a C-terminal truncated form of CRY (Fogle et al., 2011). This response remains relatively poorly resolved as it has not yet been examined using evoked potential analysis of membrane depolarization, a method that shows greater kinetic details of light evoked electrophysiological responses (Baik et al., 2019a; Au et al., 2022). The other CRY signaling mechanism involves inter-protein redox transfer for which the voltage-gated potassium beta subunit acts as a redox sensor and couple light activated CRY to changes in potassium channel activity (Fogle et al., 2015; Baik et al., 2017, 2018, 2019b; Hong et al., 2018). CRY phototransduction is mediated by light-evoked changes in the FAD redox state from an oxidized base state that absorbs UV (365 nm peak) and blue-light (450 nm) peak to its FAD•- anionic semiquinone semi-reduced state that also absorbs UV (Berndt et al., 2007; Bouly et al., 2007; Hoang et al., 2008; Öztürk et al., 2008; Lin et al., 2018). Photoactivation of the CRY FAD•- anionic semiquinone semi-reduced state yields the FADH• neutral radical state (Liu et al., 2010) which absorbs a broad peak between 580–640 nm (yellow to red) and a sharper peak at 325 nm (UV). We have yet to explore CRY physiological light responses to 325 nm UV light. Red-light photoactivation of the CRY FADH• neutral radical state is best characterized in plant CRYs, but more recent work shows that insect CRYs are also physiologically activated by red-light. This indicates that the CRY FADH• neutral radical state occurs *in vivo* (Öztürk et al., 2008; Baik et al., 2019a; Au et al., 2022). Most of the biophysical work done on the spectral absorbance properties of insect CRY proteins uses purified protein preparations. It appears that purified insect wild type CRYs do not absorb red-light when not in native cellular conditions (Berndt et al., 2007; Ozturk et al., 2011, 2014; Vaidya et al., 2013; Lin et al., 2018). It remains to be determined whether downstream signaling proteins like voltage-gated potassium subunits contribute further to species-specific differences in mosquito physiological light responses.

An alternative hypothesis for species-specific physiological light responses is based on comparative neuroanatomical analysis of diurnal *Ae. aegypti* mosquitoes and nocturnal *An. coluzzii* mosquitoes, differences in species-specific neural circuits, including PDF and PER expressing neurons may dictate attraction/avoidance behavioral light responses. Using antibodies against the well conserved PDF and PER proteins, which cross-react across a wide range of insect species, there are both similar and species-distinct features of PDF and PER expressing neural circuits of *Ae. aegypti* and *An. coluzzii* mosquitoes. PDF and PER proteins are co-expressed in the ventral lateral area in both *Ae. aegypti* and *An. coluzzii* mosquito female adult brains that can be identified as large- (l-LNvs) and small-ventral lateral neurons (s-LNvs) based on their morphological projections common to the very well characterized brains of *Drosophila melanogaster* and other insect species (Baik et al., 2020). These include the large PDF + neuronal arbors in the optic lobes that likely project from the l-LNvs and PDF + dorsal projections to the putative dorsal neurons (DNs) that likely project from the s-LNvs for both mosquito species (Renn et al., 1999; Baik et al., 2020). There are noteworthy differences between *Ae. aegypti* and *An. coluzzii* mosquito female adult brains for their PDF and PER neural circuits, notably that for *An. coluzzii*, PDF + putative s-LNv dorsal projections continue medially to the pars intercerebralis (PI) region, a major neuroendocrine center in insect brains (de Velasco et al., 2007). The PI region integrates feeding and circadian information in insulin-like peptide expressing PI neurons (Barber et al., 2016). In contrast, this distinct s-LNv to PI neural projection is absent in *Ae. aegypti* mosquito female adult brains (Baik et al., 2020). Another species-specific difference between *Ae. aegypti* and *An. coluzzii* mosquitoes is a midline crossing contralateral projection of PDF + putative l-LNvs that is detected in *An. coluzzii* mosquito female adult brains, but is not detected in *Ae. aegypti* adult female brains (Baik et al., 2020). There are entire neuronal groups that can be found in one mosquito species but not the other, notably ∼5 PER + /PDF- neurons that are detected in the medial-anterior region of *Ae. aegypti* female brains but are not seen in *An. coluzzii* mosquito female adult brains (Baik et al., 2020). Reciprocally, there are ∼7 PER + /PDF- neurons in the PI region in *An. coluzzii* that are not detected in *Ae. aegypti* (Baik et al., 2020). These similarities and differences in diurnal vs. nocturnal mosquito PDF and PER expressing neural circuits are intriguing and while we cannot yet determine at present how much they may contribute to attraction/avoidance behavioral light responses; the results herein indicate that CRY1s themselves are sufficient to confer similar species-specific light responses observed in behaving mosquitoes. It would be interesting to express diurnal *Aedes* mosquito CRY1 in a nocturnal *Anopheles* mosquito and see how this transgenic mosquito behaves in response to different light wavelengths using the light attraction/avoidance assay, along with the reciprocal experiment of expressing nocturnal *Anopheles* CRY1 in diurnal *Aedes* mosquitoes.

These findings have interesting implications for evolutionary aspects of behavior and speciation. Many insects express two forms of CRY: light sensitive CRY1s and light insensitive CRY2s which act as transcriptional repressors (Yuan et al., 2007). The evolutionary divergence between CRY1s and CRY2s appear to have occurred prior to the Cambrian radiation as multiple *cry* genes are found in sponges, an early metazoan that precedes the evolution of animal opsins (Rivera et al., 2010). Different mosquito species have evolved distinct circadian timing of behaviors according to their temporal/ecological niches, including diurnal (*Ae. aegypti*) and nocturnal (*An. coluzzii*). Numerous mosquito species-specific behaviors change with time-of-day, including flight activity, mating, oviposition, and biting (Bidlingmayer, 1994; Ditzen et al., 2008). Such behaviors enforce speciation (Wilson, 1975). Due to their large impact on health and ecology, more work on the basis of diurnality/nocturnality, behavioral timing and how species-specific niches are enforced in mosquitoes is merited.

## Data availability statement

The raw data supporting the conclusions of this article will be made available by the authors, without undue reservation.

## Author contributions

DA and TH designed research, wrote, reviewed, and edited the manuscript. DA, JL, TN AF, and SP performed research. DA, JL, AF, SP, MD, and ZY analyzed data. All authors contributed to the article and approved the submitted version.

## References

[B1] Alonso San Alberto D. Rusch C. Zhan Y. Straw A. D. Montell C. Riffell J. A. (2022). The olfactory gating of visual preferences to human skin and visible spectra in mosquitoes. *Nat. Commun.* 13:555. 10.1038/s41467-022-28195-x 35121739 PMC8816903

[B2] Au D. D. Foden A. J. Park S. J. Nguyen T. H. Liu J. C. Tran M. D. (2022). Mosquito cryptochromes expressed in *Drosophila* confer species-specific behavioral light responses. *Curr. Biol.* 32 3731–3744.e4. 10.1016/j.cub.2022.07.021 35914532 PMC9810238

[B3] Baik L. S. Recinos Y. Chevez J. A. Au D. D. Holmes T. C. (2019b). Multiple phototransduction inputs integrate to mediate UV light–evoked avoidance/attraction behavior in *Drosophila*. *J. Biol. Rhythms* 34 391–400. 10.1177/0748730419847339 31140349 PMC7118705

[B4] Baik L. S. Au D. D. Nave C. Foden A. J. Enrriquez-Villalva W. K. Holmes T. C. (2019a). Distinct mechanisms of *Drosophila* CRYPTOCHROME-mediated light-evoked membrane depolarization and in vivo clock resetting. *Proc. Natl. Acad. Sci. U.S.A.* 116 23339–23344. 10.1073/pnas.1905023116 31659046 PMC6859314

[B5] Baik L. S. Fogle K. J. Roberts L. Galschiodt A. M. Chevez J. A. Recinos Y. (2017). CRYPTOCHROME mediates behavioral executive choice in response to UV light. *Proc. Natl. Acad. Sci. U.S.A.* 114 776–781. 10.1073/pnas.1607989114 28062690 PMC5278478

[B6] Baik L. S. Nave C. Au D. D. Guda T. Chevez J. A. Ray A. (2020). Circadian regulation of light-evoked attraction and avoidance behaviors in daytime- versus nighttime-biting mosquitoes. *Curr. Biol.* 30 3252–3259.e3. 10.1016/j.cub.2020.06.010 32619483 PMC7438308

[B7] Baik L. S. Recinos Y. Chevez J. A. Holmes T. C. (2018). Circadian modulation of light-evoked avoidance/attraction behavior in *Drosophila*. *PLoS One* 13:e0201927. 10.1371/journal.pone.0201927 30106957 PMC6091921

[B8] Barber A. F. Erion R. Holmes T. C. Sehgal A. (2016). Circadian and feeding cues integrate to drive rhythms of physiology in *Drosophila* insulin-producing cells. *Genes Dev.* 30 2596–2606. 10.1101/gad.288258.116 27979876 PMC5204352

[B9] Benito J. Houl J. H. Roman G. W. Hardin P. E. (2008). The blue-light photoreceptor CRYPTOCHROME is expressed in a subset of circadian oscillator neurons in the *Drosophila* CNS. *J. Biol. Rhythms* 23 296–307. 10.1177/0748730408318588 18663237 PMC2536721

[B10] Benjamini Y. Hochberg Y. (1995). Controlling the false discovery rate: A practical and powerful approach to multiple testing. *J. R. Stat. Soc. Ser. B Methodol.* 57 289–300. 10.1111/j.2517-6161.1995.tb02031.x

[B11] Berndt A. Kottke T. Breitkreuz H. Dvorsky R. Hennig S. Alexander M. (2007). A novel photoreaction mechanism for the circadian blue light photoreceptor *Drosophila* cryptochrome. *J. Biol. Chem.* 282 13011–13021. 10.1074/jbc.M608872200 17298948

[B12] Bidlingmayer W. L. (1994). How mosquitoes see traps: Role of visual responses. *J. Am. Mosq. Control Assoc.* 10 272–279. 8965079

[B13] Bouly J.-P. Schleicher E. Dionisio-Sese M. Vandenbussche F. Van Der Straeten D. Bakrim N. (2007). Cryptochrome blue light photoreceptors are activated through interconversion of flavin redox states. *J. Biol. Chem.* 282 9383–9391. 10.1074/jbc.M609842200 17237227

[B14] Buhl E. Bradlaugh A. Ogueta M. Chen K.-F. Stanewsky R. Hodge J. J. L. (2016). Quasimodo mediates daily and acute light effects on *Drosophila* clock neuron excitability. *Proc. Natl. Acad. Sci. U.S.A.* 113 13486–13491. 10.1073/pnas.1606547113 27821737 PMC5127355

[B15] Buhl E. Higham J. P. Hodge J. J. L. (2019). Alzheimer’s disease-associated tau alters *Drosophila* circadian activity, sleep and clock neuron electrophysiology. *Neurobiol. Dis.* 130:104507. 10.1016/j.nbd.2019.104507 31207389

[B16] Busza A. Emery-Le M. Rosbash M. Emery P. (2004). Roles of the two *Drosophila* CRYPTOCHROME structural domains in circadian photoreception. *Science* 304 1503–1506. 10.1126/science.1096973 15178801

[B17] Cao G. Nitabach M. N. (2008). Circadian control of membrane excitability in *Drosophila melanogaster* lateral ventral clock neurons. *J. Neurosci.* 28 6493–6501. 10.1523/JNEUROSCI.1503-08.2008 18562620 PMC2680300

[B18] Chandrasekaran S. Schneps C. M. Dunleavy R. Lin C. DeOliveira C. C. Ganguly A. (2021). Tuning flavin environment to detect and control light-induced conformational switching in *Drosophila* cryptochrome. *Commun. Biol.* 4:249. 10.1038/s42003-021-01766-2 33637846 PMC7910608

[B19] Chaturvedi R. Stork T. Yuan C. Freeman M. R. Emery P. (2022). Astrocytic GABA transporter controls sleep by modulating GABAergic signaling in *Drosophila* circadian neurons. *Curr. Biol.* 32 1895–1908.e5. 10.1016/j.cub.2022.02.066 35303417 PMC9090989

[B20] Chiu J. C. Low K. H. Pike D. H. Yildirim E. Edery I. (2010). Assaying locomotor activity to study circadian rhythms and sleep parameters in *Drosophila*. *J. Vis. Exp.* 43:2157. 10.3791/2157 20972399 PMC3229366

[B21] Coombe P. E. (1982). Visual behaviour of the greenhouse whitefly, *Trialeurodes vaporariorum*. *Physiol. Entomol.* 7 243–251. 10.1111/j.1365-3032.1982.tb00297.x

[B22] Damulewicz M. Mazzotta G. M. (2020). One actor, multiple roles: The performances of cryptochrome in *Drosophila*. *Front. Physiol.* 11:99. 10.3389/fphys.2020.00099 32194430 PMC7066326

[B23] Das S. Dimopoulos G. (2008). Molecular analysis of photic inhibition of blood-feeding in *Anopheles gambiae*. *BMC Physiol.* 8:23. 10.1186/1472-6793-8-23 19087335 PMC2646746

[B24] de Velasco B. Erclik T. Shy D. Sclafani J. Lipshitz H. McInnes R. (2007). Specification and development of the pars intercerebralis and pars lateralis, neuroendocrine command centers in the *Drosophila* brain. *Dev. Biol.* 302 309–323. 10.1016/j.ydbio.2006.09.035 17070515

[B25] Dissel S. Codd V. Fedic R. Garner K. J. Costa R. Kyriacou C. P. (2004). A constitutively active cryptochrome in *Drosophila melanogaster*. *Nat. Neurosci.* 7 834–840. 10.1038/nn1285 15258584

[B26] Ditzen M. Pellegrino M. Vosshall L. B. (2008). Insect odorant receptors are molecular targets of the insect repellent DEET. *Science* 319 1838–1842. 10.1126/science.1153121 18339904

[B27] Emery P. So W. V. Kaneko M. Hall J. C. Rosbash M. (1998). CRY, a *Drosophila* clock and light-regulated cryptochrome, is a major contributor to circadian rhythm resetting and photosensitivity. *Cell* 95 669–679. 10.1016/S0092-8674(00)81637-2 9845369

[B28] Emery P. Stanewsky R. Helfrich-Förster C. Emery-Le M. Hall J. C. Rosbash M. (2000). *Drosophila* CRY Is a deep brain circadian photoreceptor. *Neuron* 26 493–504. 10.1016/S0896-6273(00)81181-2 10839367

[B29] Farnesi L. C. Barbosa C. S. Araripe L. O. Bruno R. V. (2018). The influence of a light and dark cycle on the egg laying activity of *Aedes aegypti* (Linnaeus, 1762) (Diptera: Culicidae). *Mem. Inst. Oswaldo Cruz* 113:e170362. 10.1590/0074-02760170362 29412343 PMC5851057

[B30] Fernandez-Chiappe F. Frenkel L. Colque C. C. Ricciuti A. Hahm B. Cerredo K. (2021). High-frequency neuronal bursting is essential for circadian and sleep behaviors in *Drosophila*. *J. Neurosci.* 41 689–710. 10.1523/JNEUROSCI.2322-20.2020 33262246 PMC7842748

[B31] Flourakis M. Allada R. (2015). “Patch-clamp electrophysiology in *Drosophila* circadian pacemaker neurons,” in *Methods in enzymology*, (Amsterdam: Elsevier), 23–44. 10.1016/bs.mie.2014.10.005 25707271

[B32] Flourakis M. Kula-Eversole E. Hutchison A. L. Han T. H. Aranda K. Moose D. L. (2015). A conserved bicycle model for circadian clock control of membrane excitability. *Cell* 162 836–848. 10.1016/j.cell.2015.07.036 26276633 PMC4537776

[B33] Fogle K. J. Baik L. S. Houl J. H. Tran T. T. Roberts L. Dahm N. A. (2015). CRYPTOCHROME-mediated phototransduction by modulation of the potassium ion channel β-subunit redox sensor. *Proc. Natl. Acad. Sci. U.S.A.* 112 2245–2250. 10.1073/pnas.1416586112 25646452 PMC4343116

[B34] Fogle K. J. Parson K. G. Dahm N. A. Holmes T. C. (2011). CRYPTOCHROME is a blue-light sensor that regulates neuronal firing rate. *Science* 331 1409–1413. 10.1126/science.1199702 21385718 PMC4418525

[B35] Gegear R. J. Foley L. E. Casselman A. Reppert S. M. (2010). Animal cryptochromes mediate magnetoreception by an unconventional photochemical mechanism. *Nature* 463 804–807. 10.1038/nature08719 20098414 PMC2820607

[B36] Giachello C. N. G. Scrutton N. S. Jones A. R. Baines R. A. (2016). Magnetic fields modulate blue-light-dependent regulation of neuronal firing by cryptochrome. *J. Neurosci.* 36 10742–10749. 10.1523/JNEUROSCI.2140-16.2016 27798129 PMC5083005

[B37] Green C. H. Cosens D. (1983). Spectral responses of the tsetse fly, *Glossina morsitans morsitans*. *J. Insect Physiol.* 29 795–800. 10.1016/0022-1910(83)90009-4

[B38] Harrisingh M. C. Wu Y. Lnenicka G. A. Nitabach M. N. (2007). Intracellular Ca2+ regulates free-running circadian clock oscillation in vivo. *J. Neurosci.* 27 12489–12499. 10.1523/JNEUROSCI.3680-07.2007 18003827 PMC6673328

[B39] Hoang N. Schleicher E. Kacprzak S. Bouly J.-P. Picot M. Wu W. (2008). Human and *Drosophila* cryptochromes are light activated by flavin photoreduction in living cells. *PLoS Biol.* 6:e160. 10.1371/journal.pbio.0060160 18597555 PMC2443192

[B40] Holmes T. C. Sheeba V. Mizrak D. Rubovszky B. Dahdal D. (2007). “Circuit-breaking and behavioral analysis by molecular genetic manipulation of neural activity in *Drosophila*,” in *Invertebrate neurobiology*, eds North G. Greenspan R. (Cold Spring Harbor, NY: Cold Spring Harbor Press), 19–52.

[B41] Hong G. Pachter R. Ritz T. (2018). Coupling *Drosophila melanogaster* cryptochrome light activation and oxidation of the Kvβ subunit hyperkinetic NADPH cofactor. *J. Phys. Chem. B* 122 6503–6510. 10.1021/acs.jpcb.8b03493 29847128

[B42] Jones M. D. R. Hill M. Hope A. M. (1967). The circadian flight activity of the mosquito, *Anopheles gambiae*?: Phase setting by the light regime. *J. Exp. Biol.* 47 503–511. 10.1242/jeb.47.3.503 5592417

[B43] Kistenpfennig C. Nakayama M. Nihara R. Tomioka K. Helfrich-Förster C. Yoshii T. (2018). A tug-of-war between cryptochrome and the visual system allows the adaptation of evening activity to long photoperiods in *Drosophila melanogaster*. *J. Biol. Rhythms* 33 24–34. 10.1177/0748730417738612 29179610

[B44] Klarsfeld A. Malpel S. Michard-Vanhée C. Picot M. Chélot E. Rouyer F. (2004). Novel features of cryptochrome-mediated photoreception in the brain circadian clock of *Drosophila*. *J. Neurosci.* 24 1468–1477. 10.1523/JNEUROSCI.3661-03.2004 14960620 PMC6730330

[B45] Knop E. Zoller L. Ryser R. Gerpe C. Hörler M. Fontaine C. (2017). Artificial light at night as a new threat to pollination. *Nature* 548 206–209. 10.1038/nature23288 28783730

[B46] Koh K. Zheng X. Sehgal A. (2006). JETLAG resets the *Drosophila* circadian clock by promoting light-induced degradation of TIMELESS. *Science* 312 1809–1812. 10.1126/science.1124951 16794082 PMC2767177

[B47] Lazopulo S. Lazopulo A. Baker J. D. Syed S. (2019). Daytime colour preference in *Drosophila* depends on the circadian clock and TRP channels. *Nature* 574 108–111. 10.1038/s41586-019-1571-y 31534223

[B48] Li M.-T. Cao L.-H. Xiao N. Tang M. Deng B. Yang T. (2018). Hub-organized parallel circuits of central circadian pacemaker neurons for visual photoentrainment in *Drosophila*. *Nat. Commun.* 9:4247. 10.1038/s41467-018-06506-5 30315165 PMC6185921

[B49] Lin C. Schneps C. M. Chandrasekaran S. Ganguly A. Crane B. R. (2022). Mechanistic insight into light-dependent recognition of timeless by *Drosophila* cryptochrome. *Struct. Lond. Engl* 30 851–861.e5. 10.1016/j.str.2022.03.010 35397203 PMC9201872

[B50] Lin C. Top D. Manahan C. C. Young M. W. Crane B. R. (2018). Circadian clock activity of cryptochrome relies on tryptophan-mediated photoreduction. *Proc. Natl. Acad. Sci. U.S.A.* 115 3822–3827. 10.1073/pnas.1719376115 29581265 PMC5899454

[B51] Liu B. Liu H. Zhong D. Lin C. (2010). Searching for a photocycle of the cryptochrome photoreceptors. *Curr. Opin. Plant Biol.* 13 578–586. 10.1016/j.pbi.2010.09.005 20943427 PMC2972227

[B52] Liu S. Lamaze A. Liu Q. Tabuchi M. Yang Y. Fowler M. (2014). WIDE AWAKE mediates the circadian timing of sleep onset. *Neuron* 82 151–166. 10.1016/j.neuron.2014.01.040 24631345 PMC3982794

[B53] McCarthy E. V. Wu Y. Decarvalho T. Brandt C. Cao G. Nitabach M. N. (2011). Synchronized bilateral synaptic inputs to *Drosophila melanogaster* neuropeptidergic rest/arousal neurons. *J. Neurosci.* 31 8181–8193. 10.1523/JNEUROSCI.2017-10.2011 21632940 PMC3125135

[B54] Muraro N. I. Ceriani M. F. (2015). Acetylcholine from visual circuits modulates the activity of arousal neurons in *Drosophila*. *J. Neurosci.* 35 16315–16327. 10.1523/JNEUROSCI.1571-15.2015 26674859 PMC6605507

[B55] Nave C. Roberts L. Hwu P. Estrella J. D. Vo T. C. Nguyen T. H. (2021). Weekend light shifts evoke persistent *Drosophila* circadian neural network desynchrony. *J. Neurosci.* 41 5173–5189. 10.1523/JNEUROSCI.3074-19.2021 33931552 PMC8211545

[B56] Ni J. D. Baik L. S. Holmes T. C. Montell C. (2017). A rhodopsin in the brain functions in circadian photoentrainment in *Drosophila*. *Nature* 545 340–344. 10.1038/nature22325 28489826 PMC5476302

[B57] Nitabach M. N. Blau J. Holmes T. C. (2002). Electrical silencing of *Drosophila* pacemaker neurons stops the free-running circadian clock. *Cell* 109 485–495. 10.1016/S0092-8674(02)00737-7 12086605

[B58] Ozturk N. Selby C. P. Annayev Y. Zhong D. Sancar A. (2011). Reaction mechanism of *Drosophila* cryptochrome. *Proc. Natl. Acad. Sci. U.S.A.* 108 516–521. 10.1073/pnas.1017093108 21187431 PMC3021015

[B59] Ozturk N. Selby C. P. Zhong D. Sancar A. (2014). Mechanism of photosignaling by *Drosophila* cryptochrome. *J. Biol. Chem.* 289 4634–4642. 10.1074/jbc.M113.542498 24379403 PMC3931024

[B60] Öztürk N. Song S.-H. Selby C. P. Sancar A. (2008). Animal type 1 cryptochromes. *J. Biol. Chem.* 283 3256–3263. 10.1074/jbc.M708612200 18056988

[B61] Padilha K. P. Resck M. E. B. Cunha O. A. T. da Teles-de-Freitas R. Campos S. S. Sorgine M. H. F. (2018). Zika infection decreases *Aedes aegypti* locomotor activity but does not influence egg production or viability. *Mem. Inst. Oswaldo Cruz* 113:e180290. 10.1590/0074-02760180290 30156598 PMC6107100

[B62] Parisky K. M. Agosto J. Pulver S. R. Shang Y. Kuklin E. Hodge J. J. L. (2008). PDF cells are a GABA-responsive wake-promoting component of the *Drosophila* sleep circuit. *Neuron* 60 672–682. 10.1016/j.neuron.2008.10.042 19038223 PMC2734413

[B63] Peschel N. Chen K. F. Szabo G. Stanewsky R. (2009). Light-dependent interactions between the *Drosophila* circadian clock factors cryptochrome, jetlag, and timeless. *Curr. Biol.* 19 241–247. 10.1016/j.cub.2008.12.042 19185492

[B64] Potdar S. Sheeba V. (2018). Wakefulness is promoted during day time by PDFR signalling to dopaminergic neurons in *Drosophila melanogaster*. *eNeuro* 5:ENEURO.0129-18.2018. 10.1523/ENEURO.0129-18.2018 30131970 PMC6102377

[B65] Renn S. C. P. Park J. H. Rosbash M. Hall J. C. Taghert P. H. (1999). A pdf neuropeptide gene mutation and ablation of PDF neurons each cause severe abnormalities of behavioral circadian rhythms in *Drosophila*. *Cell* 99 791–802. 10.1016/S0092-8674(00)81676-1 10619432

[B66] Rivera A. S. Pankey M. S. Plachetzki D. C. Villacorta C. Syme A. E. Serb J. M. (2010). Gene duplication and the origins of morphological complexity in pancrustacean eyes, a genomic approach. *BMC Evol. Biol.* 10:123. 10.1186/1471-2148-10-123 20433736 PMC2888819

[B67] Rund S. S. C. Lee S. J. Bush B. R. Duffield G. E. (2012). Strain- and sex-specific differences in daily flight activity and the circadian clock of *Anopheles gambiae* mosquitoes. *J. Insect Physiol.* 58 1609–1619. 10.1016/j.jinsphys.2012.09.016 23068991

[B68] Sakai K. Tsutsui K. Yamashita T. Iwabe N. Takahashi K. Wada A. (2017). *Drosophila melanogaster* rhodopsin Rh7 is a UV-to-visible light sensor with an extraordinarily broad absorption spectrum. *Sci. Rep.* 7:7349. 10.1038/s41598-017-07461-9 28779161 PMC5544684

[B69] Sawadogo S. P. Costantini C. Pennetier C. Diabaté A. Gibson G. Dabiré R. K. (2013). Differences in timing of mating swarms in sympatric populations of *Anopheles coluzzii* and *Anopheles gambiae* s.s. (formerly *An. gambiae* M and S molecular forms) in Burkina Faso. *West Africa. Parasit. Vectors* 6:275. 10.1186/1756-3305-6-275 24330578 PMC3851435

[B70] Schlichting M. Menegazzi P. Helfrich-Förster C. (2015). Normal vision can compensate for the loss of the circadian clock. *Proc. R. Soc. B Biol. Sci. U.S.A.* 282:20151846. 10.1098/rspb.2015.1846 26378222 PMC4614763

[B71] Seluzicki A. Flourakis M. Kula-Eversole E. Zhang L. Kilman V. Allada R. (2014). Dual PDF signaling pathways reset clocks via TIMELESS and acutely excite target neurons to control circadian behavior. *PLoS Biol.* 12:e1001810. 10.1371/journal.pbio.1001810 24643294 PMC3958333

[B72] Shang Y. Griffith L. C. Rosbash M. (2008). Light-arousal and circadian photoreception circuits intersect at the large PDF cells of the *Drosophila* brain. *Proc. Natl. Acad. Sci. U.S.A.* 105 19587–19594. 10.1073/pnas.0809577105 19060186 PMC2596742

[B73] Sheeba V. Fogle K. J. Holmes T. C. (2010). Persistence of morning anticipation behavior and high amplitude morning startle response following functional loss of small ventral lateral neurons in *Drosophila*. *PLoS One* 5:e11628. 10.1371/journal.pone.0011628 20661292 PMC2905440

[B74] Sheeba V. Sharma V. K. Gu H. Chou Y.-T. O’Dowd D. K. Holmes T. C. (2008c). Pigment dispersing factor-dependent and -independent circadian locomotor behavioral rhythms. *J. Neurosci.* 28 217–227. 10.1523/JNEUROSCI.4087-07.2008 18171939 PMC2692917

[B75] Sheeba V. Gu H. Sharma V. K. O’Dowd D. K. Holmes T. C. (2008b). Circadian- and light-dependent regulation of resting membrane potential and spontaneous action potential firing of *Drosophila* circadian pacemaker neurons. *J. Neurophysiol.* 99 976–988. 10.1152/jn.00930.2007 18077664 PMC2692874

[B76] Sheeba V. Fogle K. J. Kaneko M. Rashid S. Chou Y.-T. Sharma V. K. (2008a). Large ventral lateral neurons modulate arousal and sleep in *Drosophila*. *Curr. Biol.* 18 1537–1545. 10.1016/j.cub.2008.08.033 18771923 PMC2597195

[B77] Sheppard A. D. Rund S. S. C. George G. F. Clark E. Acri D. J. Duffield G. E. (2017). Light manipulation of mosquito behaviour: Acute and sustained photic suppression of biting activity in the *Anopheles gambiae* malaria mosquito. *Parasit. Vectors* 10:255. 10.1186/s13071-017-2196-3 28619089 PMC5472875

[B78] Smith P. Buhl E. Tsaneva-Atanasova K. Hodge J. J. L. (2019). Shaw and Shal voltage-gated potassium channels mediate circadian changes in *Drosophila* clock neuron excitability. *J. Physiol.* 597 5707–5722. 10.1113/JP278826 31612994

[B79] Stanewsky R. Kaneko M. Emery P. Beretta B. Wager-Smith K. Kay S. A. (1998). The cryb mutation identifies cryptochrome as a circadian photoreceptor in *Drosophila*. *Cell* 95 681–692. 10.1016/S0092-8674(00)81638-4 9845370

[B80] Sumba L. A. Okoth K. Deng A. L. Githure J. Knols B. G. J. Beier J. C. (2004). Daily oviposition patterns of the African malaria mosquito *Anopheles gambiae* Giles (Diptera: Culicidae) on different types of aqueous substrates. *J. Circadian Rhythms* 2:6. 10.1186/1740-3391-2-6 15596009 PMC544589

[B81] Tabuchi M. Coates K. E. Bautista O. B. Zukowski L. H. (2021). Light/clock influences membrane potential dynamics to regulate sleep states. *Front. Neurol.* 12:625369. 10.3389/fneur.2021.625369 33854471 PMC8039321

[B82] Taylor B. Jones M. D. R. (1969). The circadian rhythm of flight activity in the mosquito *Aedes aegypti* (L.): The phase-setting effects of light-on and light-off. *J. Exp. Biol.* 51 59–70. 10.1242/jeb.51.1.59 5387705

[B83] Tokushima Y. Uehara T. Yamaguchi T. Arikawa K. Kainoh Y. Shimoda M. (2016). Broadband photoreceptors are involved in violet light preference in the parasitoid fly *Exorista japonica*. *PLoS One* 11:e0160441. 10.1371/journal.pone.0160441 27532635 PMC4988788

[B84] Vaidya A. T. Top D. Manahan C. C. Tokuda J. M. Zhang S. Pollack L. (2013). Flavin reduction activates *Drosophila* cryptochrome. *Proc. Natl. Acad. Sci. U.S.A.* 110 20455–20460. 10.1073/pnas.1313336110 24297896 PMC3870761

[B85] Wilson E. O. (1975). *Sociobiology: The new synthesis.* Cambridge, MA: Harvard University Press.

[B86] Yamaguchi S. Desplan C. Heisenberg M. (2010). Contribution of photoreceptor subtypes to spectral wavelength preference in *Drosophila*. *Proc. Natl. Acad. Sci. U.S.A.* 107 5634–5639. 10.1073/pnas.0809398107 20212139 PMC2851746

[B87] Yoshii T. Todo T. Wülbeck C. Stanewsky R. Helfrich-Förster C. (2008). Cryptochrome is present in the compound eyes and a subset of *Drosophila*’s clock neurons. *J. Comp. Neurol.* 508 952–966. 10.1002/cne.21702 18399544

[B88] Yuan Q. Metterville D. Briscoe A. D. Reppert S. M. (2007). Insect cryptochromes: Gene duplication and loss define diverse ways to construct insect circadian clocks. *Mol. Biol. Evol.* 24 948–955. 10.1093/molbev/msm011 17244599

[B89] Zhao J. Kilman V. L. Keegan K. P. Peng Y. Emery P. Rosbash M. (2003). *Drosophila* clock can generate ectopic circadian clocks. *Cell* 113 755–766. 10.1016/S0092-8674(03)00400-8 12809606

