# Erratum: Nocturnal mosquito Cryptochrome 1 mediates greater electrophysiological and behavioral responses to blue light relative to diurnal mosquito Cryptochrome 1

**DOI:** 10.3389/fnins.2022.1129968

**Published:** 2023-01-09

**Authors:** Frontiers Production Office

**Affiliations:** Frontiers Media SA, Lausanne, Switzerland

**Keywords:** cryptochrome, non-image forming vision, electrophysiology, light-evoked behavior, mosquito sensory biology, *Drosophila melanogaster*, *Anopheles gambiae*, *Aedes aegypti*

Due to a production error, the contribution of junior reviewer Florencia Fernandez-Chiappe was omitted. It should appear as follows: Florencia Fernandez-Chiappe CONICET Instituto de Investigación en Biomedicina de Buenos Aires (IBioBA), Argentina, in collaboration with reviewer NM.

The publisher apologizes for this mistake. The original version of this article has been updated.

