# Supplementary material for: Nocturnal mosquito Cryptochrome 1 mediates greater electrophysiological and behavioral responses to blue light relative to diurnal mosquito Cryptochrome 1
Source: Front Neurosci. 2022 Nov 30;16:1042508. doi: 10.3389/fnins.2022.1042508 (PMC9749892; doi:10.3389/fnins.2022.1042508)
Supplement: Supplementary file 1 [file Data_Sheet_1.pdf]

A

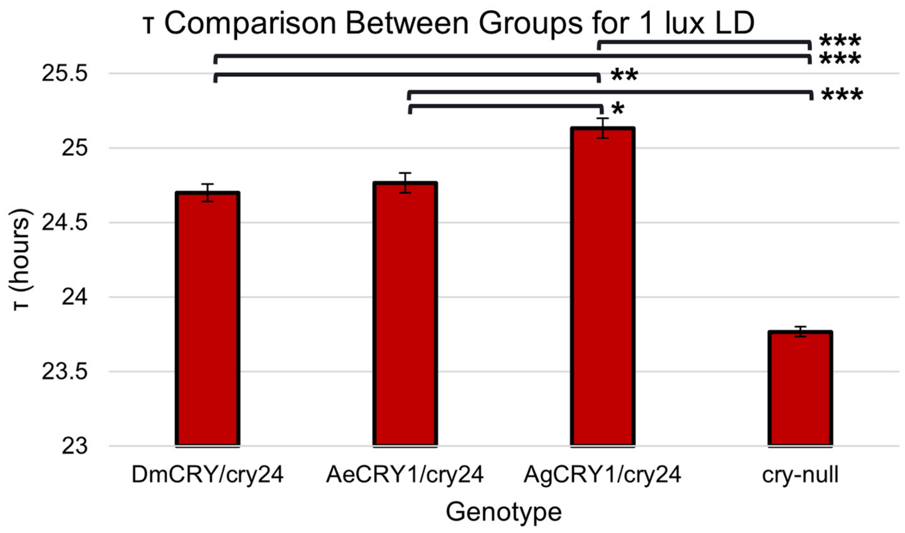

B

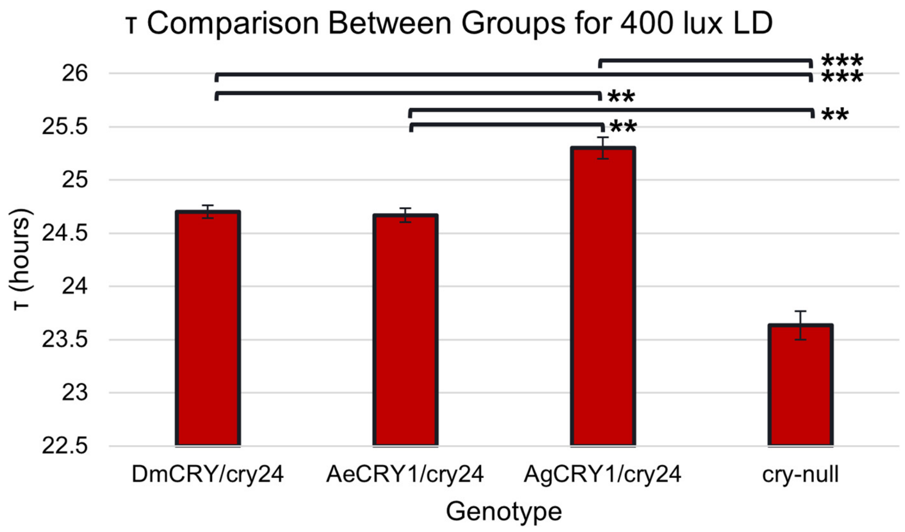

Supplementary Figure 1. Comparison of period length during 1 lux and 400 lux LD entrainment

Average period length (tau, τ) over 5 days of LD entrainment for DmCRY, AeCRY1, AgCRY1, and *cry-null* groups are quantified for (**A**) 1 lux and (**B**) 400 lux light intensity. Pairwise t-tests were performed for statistical comparison. Data are represented as mean ± SEM. One significance symbol; $p \leq 0.05$, two significance symbols; $p \leq 0.005$, three significance symbols; $p \leq 0.001$.

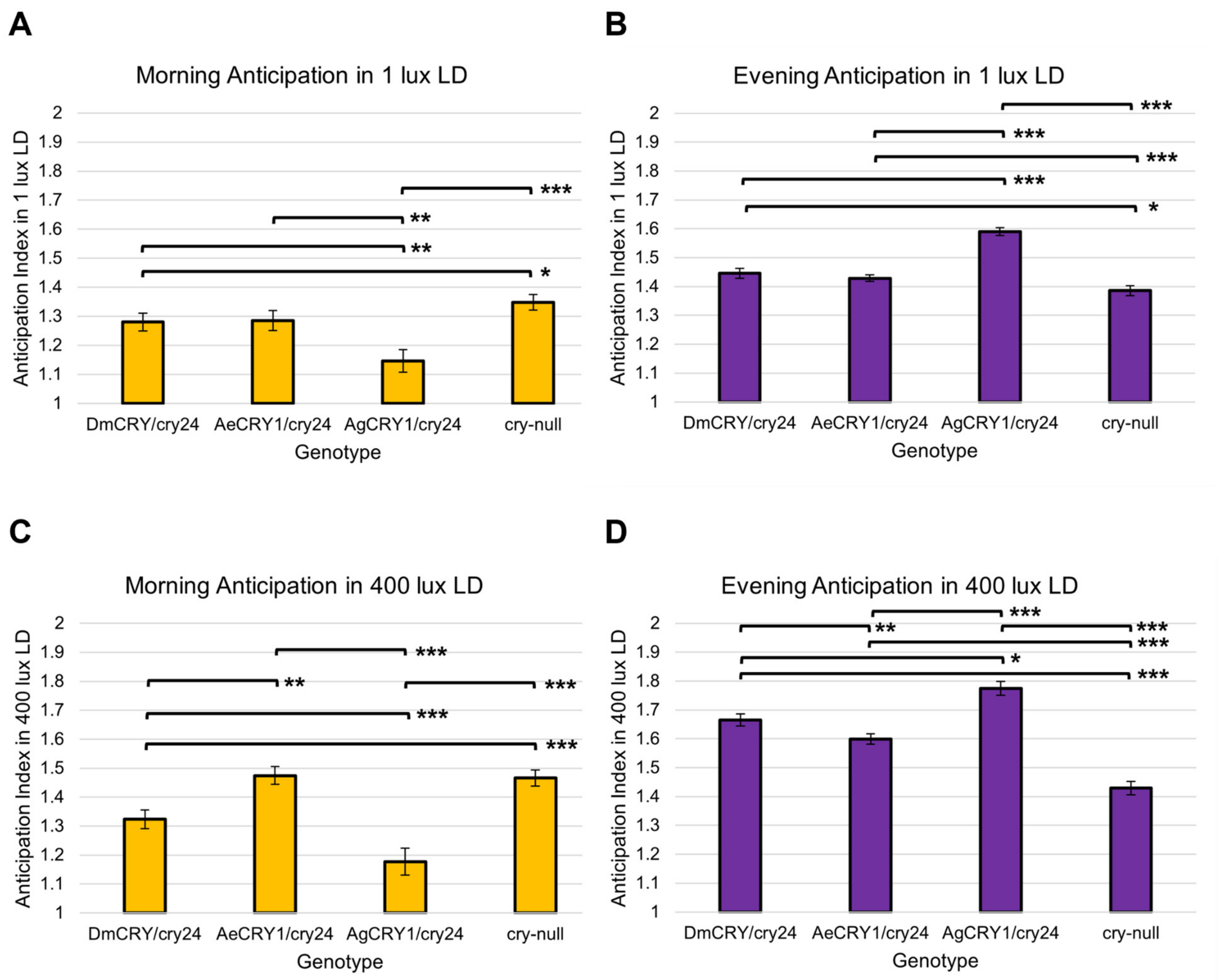

Supplementary Figure 2. Morning and evening anticipatory indices for DmCRY, AeCRY1, AgCRY1, and *cry-null* at 1 lux and 400 lux LD

Quantification of morning and evening anticipation indices for (**A, B**) low 1 lux LD, and (**C, D**) moderately high 400 lux LD entrainment. (**A, C**) morning and (**B, D**) evening anticipation indices were measured as the ratio of average activity three hours before lights on or off to the average activity six hours before lights on or off, respectively. Pairwise t-tests were performed for statistical comparison. Data are represented as mean ± SEM. One significance symbol; $p \leq 0.05$, two significance symbols; $p \leq 0.005$, three significance symbols; $p \leq 0.001$.
